# Partial Pressure of Arterial Oxygen in Healthy Adults at High Altitudes: A Systematic Review and Meta-Analysis

**DOI:** 10.1001/jamanetworkopen.2023.18036

**Published:** 2023-06-16

**Authors:** Aglaia Forrer, Thomas Gaisl, Ahmet Sevik, Michelle Meyer, Luzi Senteler, Mona Lichtblau, Konrad Ernst Bloch, Silvia Ulrich, Michael Furian

**Affiliations:** 1 Department of Respiratory Medicine, University Hospital Zurich, Zurich, Switzerland; 2 Department of Epidemiology, Harvard T.H. Chan School of Public Health, Boston, Massachusetts; 3 Swiss-Kyrgyz High-Altitude Medicine and Research Initiative, Zurich, Switzerland; 4 Research Department, Swiss University for Traditional Chinese Medicine, Bad Zurzach, Switzerland

## Abstract

**Importance:**

With increasing altitude, the partial pressure of inspired oxygen decreases and, consequently, the Pao_2_ decreases. Even though this phenomenon is well known, the extent of the reduction as a function of altitude remains unknown.

**Objective:**

To calculate an effect size estimate for the decrease in Pao_2_ with each kilometer of vertical gain among healthy unacclimatized adults and to identify factors associated with Pao_2_ at high altitude (HA).

**Data Sources:**

A systematic search of PubMed and Embase was performed from database inception to April 11, 2023. Search terms included *arterial blood gases* and *altitude*.

**Study Selection:**

A total of 53 peer-reviewed prospective studies in healthy adults providing results of arterial blood gas analysis at low altitude (<1500 m) and within the first 3 days at the target altitude (≥1500 m) were analyzed.

**Data Extraction and Synthesis:**

Primary and secondary outcomes as well as study characteristics were extracted from the included studies, and individual participant data (IPD) were requested. Estimates were pooled using a random-effects DerSimonian-Laird model for the meta-analysis.

**Main Outcomes and Measures:**

Mean effect size estimates and 95% CIs for reduction in Pao_2_ at HA and factors associated with Pao_2_ at HA in healthy adults.

**Results:**

All of the 53 studies involving 777 adults (mean [SD] age, 36.2 [10.5] years; 510 men [65.6%]) reporting 115 group ascents to altitudes between 1524 m and 8730 m were included in the aggregated data analysis; 13 of those studies involving 305 individuals (mean [SD] age, 39.8 [13.6] years; 185 men [60.7%]) reporting 29 ascents were included in the IPD analysis. The estimated effect size of Pao_2_ was −1.60 kPa (95% CI, −1.73 to −1.47 kPa) for each 1000 m of altitude gain (τ^2^ = 0.14; *I*^2^ = 86%). The Pao_2_ estimation model based on IPD data revealed that target altitude (−1.53 kPa per 1000 m; 95% CI, −1.63 to −1.42 kPa per 1000 m), age (−0.01 kPa per year; 95% CI, −0.02 to −0.003 kPa per year), and time spent at an altitude of 1500 m or higher (0.16 kPa per day; 95% CI, 0.11-0.21 kPa per day) were significantly associated with Pao_2_.

**Conclusions and Relevance:**

In this systematic review and meta-analysis, the mean decrease in Pao_2_ was 1.60 kPa per 1000 m of vertical ascent. This effect size estimate may improve the understanding of physiological mechanisms, assist in the clinical interpretation of acute altitude illness in healthy individuals, and serve as a reference for physicians counseling patients with cardiorespiratory disease who are traveling to HA regions.

## Key Points

**Question:**

What is the effect size estimation for the decrease in Pao_2_ in healthy unacclimatized adults when traveling to high-altitude (HA) regions, and what factors are associated with Pao_2_ at HA?

**Findings:**

In this systematic review and meta-analysis including 53 prospective studies and 777 adult participants ascending to HA between 1524 m and 8730 m, the mean decrease in Pao_2_ was 1.60 kPa for each kilometer of altitude gain. Target altitude (≥1500 m), age, and time spent at target altitude were significantly associated with Pao_2_.

**Meaning:**

This study’s finding of a Pao_2_ effect size estimate in healthy individuals may improve the understanding of physiological mechanisms, assist in clinical interpretation of acute altitude illness in healthy individuals, and serve as a reference for physicians counseling patients with cardiorespiratory disease who are traveling to HA regions.

## Teaser

This systematic review and meta-analysis provides an effect size estimate for the decrease in Pao_2_ with each kilometer of vertical gain and assesses factors associated with Pao_2_ at high altitudes among healthy unacclimatized adults.

## Introduction

With the improvement of infrastructure and means of transportation, 200 million people are estimated to visit regions at altitudes higher than 1500 m each year, be it for recreational or professional activities.^1^ However, travel to high-altitude (HA) regions imposes several challenges on the human body because with the increase in altitude, the barometric pressure (Pb) decreases, with proportional reductions in the pressure of inspired oxygen (Pio_2_). This process entails a decrease in Pao_2_,^2^ which in turn leads to hypoxemia and a series of physiological mechanisms counteracting the decrease of Pao_2_.^3^

For healthy but unacclimatized individuals permanently living at an altitude lower than 1500 m, acute ascents to altitudes between 2000 m and 2500 m (equivalent to pressurized airplane cabins during long-distance flights)^1^ are readily tolerated, whereas fast ascents to altitudes higher than 2500 m can cause an individual and highly variable degree of hypoxemia, sleep disturbance,^4,5^ exercise intolerance,^6^ and acute mountain sickness (AMS).^7^

To better understand the physiological changes and health problems occurring at HA, the decrease in Pb (and therefore reduction in inspired oxygen) is an obvious target for investigation because it is unique to HA. With the invention of arterial blood gas (ABG) analysis in 1957,^8^ a new possibility emerged to measure the consequences of the lower PiO_2_ for the body. Soon after the invention of ABG analysis, the first measurements of Pao_2_ at HA had been performed.^9^ Since those early days, many more studies including ABG analyses at HA have been performed, but results from these analyses were mostly secondary outcomes. Reference values for ABG at HA were only published for HA native populations^10^ but not for low-altitude (LA) populations traveling to HA regions.

Although hypoxemia at HA is well documented, no study to date has comprehensively quantified Pao_2_ changes in response to a wide range of altitudes. This knowledge is important to improve physiological and clinical understanding of the interindividual variability of acute HA tolerance and illnesses in healthy individuals. Moreover, reference values and CIs for healthy individuals would provide guidance when counseling patients with preexisting diseases who are planning a sojourn to an HA region and when providing treatment for those patients while they are at HA. Therefore, the purpose of this systematic review and meta-analysis was to calculate an effect size estimate for the decrease in Pao_2_ with each kilometer of vertical gain and to assess the factors associated with Pao_2_ at HA in healthy unacclimatized people traveling to HA regions.

## Methods

This systematic review and meta-analysis followed the Preferred Reporting Items for Systematic Reviews and Meta-Analyses (PRISMA) reporting guideline.^11^ The study was registered in PROSPERO (CRD42021283236). All included studies received ethical review, and all participants provided informed consent.

### Literature Search

PubMed (Bookshelf, MEDLINE, and PubMed Central) and Embase were systematically searched for peer-reviewed articles from database inception to April 11, 2023. Search terms included *arterial blood gases* and *altitude* (a full list of search terms is provided in the eMethods in Supplement 1). Titles and abstracts of the records were screened, and the full texts were obtained if they met the inclusion criteria.

### Data Extraction

Data were extracted by 1 investigator (A.F.) using a standardized prepiloted form from all studies meeting eligibility criteria for inclusion. When uncertainties about eligibility arose, a second investigator (M.F.) was involved, and the question was solved through discussion. If several articles reported data for the same cohort, the article with the most complete data was chosen, and additional information was added to this entry. To be able to conduct an individual participant data (IPD) analysis, additional data for the included studies were requested from the first, last, or corresponding authors specified in the article.

### Study Selection Criteria

Arterial blood gas measurements by arterial puncture or catheter (not capillary) at altitudes of 1500 m or higher or hypobaric chambers depressurized to altitudes of 1500 m or higher were compulsory, as were baseline measurements in the same cohort at altitudes lower than 1500 m. For our systematic review, we only considered studies measuring the acute consequences of HA for participants; therefore, only ABG measurements taken within the first 72 hours at the target altitude were included. Studies involving HA native populations, participants susceptible to altitude-related illnesses, or participants receiving medical prophylaxis were excluded, as were studies with an unclear ascent protocol. Only prospective studies published in a journal written in the English, French, or German language were considered.

### Outcomes

The primary outcome was the Pao_2_ in healthy adults traveling to altitudes of 1500 m or higher within the first 72 hours at the target altitude. Secondary outcomes were other ABG parameters, including Paco_2_, arterial oxygen saturation (Sao_2_), and pH. When a study reported the incidence of altitude-related adverse health effects including AMS, the data were retrieved. We collected baseline demographic information, including age, sex, and body mass index (BMI; calculated as weight in kilograms divided by height in meters squared). Additional information was retrieved as outlined in the eMethods in Supplement 1.

### Study Risk of Bias Assessment

Two researchers (M.M. and L.S.) assessed the risk of bias independently using the Quality Assessment Tool for Observational Cohort and Cross-Sectional Studies.^12^ The tool is composed of 14 individual questions, each answered with yes, no, or not reported if no answer could be retrieved within the study. The studies were classified according to the system used by Bagias et al^13^ and as outlined in the eMethods in Supplement 1.

### Statistical Analysis

Detailed statistical considerations are outlined in the eMethods in Supplement 1. Arterial blood gas outcomes were analyzed using linear regression models adjusted for altitude. The a priori linearity assumption was tested for independence, linearity, homoscedasticity of the residuals, and normality of the residuals. The data were then converted to the altitude effect. The mean effect was calculated as the difference in kPa divided by the difference in meters, then multiplied by 1000. The SE of the mean was calculated as follows:

(SE*_l_*^2^ + SE*_h_*^2^)^1/2^,

where *l* is low altitude and *h* is high altitude. Because a subgroup of 19 studies^1,3,6,7,10,13,14,15,16,17,18,19,20,21,22,23,24,25,26^ published data on multiple assessments (range, 2-8 assessments) performed in the same individuals, a fixed-effects intrastudy multivariate meta-analysis^14^ was performed to account for the dependence among the outcomes. There was no evidence for significant heterogeneity between assessments, and the calculated mean estimate of the correlation (*r*) between ascents was 0.53.

The calculated overall estimate of each study was then incorporated into an interstudy DerSimonian-Laird random-effects model for multivariate meta-analysis. Heterogeneity was assessed using the estimated between-study variance (τ^2^) and the *I*^2^ statistic. An estimation model for Pao_2_ at HA was developed by a stepwise forward approach using mixed regression analysis and based on baseline characteristics of IPD. Due to the potential exponential association between the primary outcome of altitude and Pao_2_, a sensitivity analysis was performed using log(Pao_2_) as the dependent variable. In the IPD analysis, the lower 5% (lower limit of normal [LLN]) and upper 95% (upper limit of normal [ULN]) CI boundaries were calculated for ABG. All statistical analyses were performed using Stata software, version 17 (StataCorp LLC). The threshold for statistical significance was 2-tailed *P* = .05.

## Results

**Figure 1.**
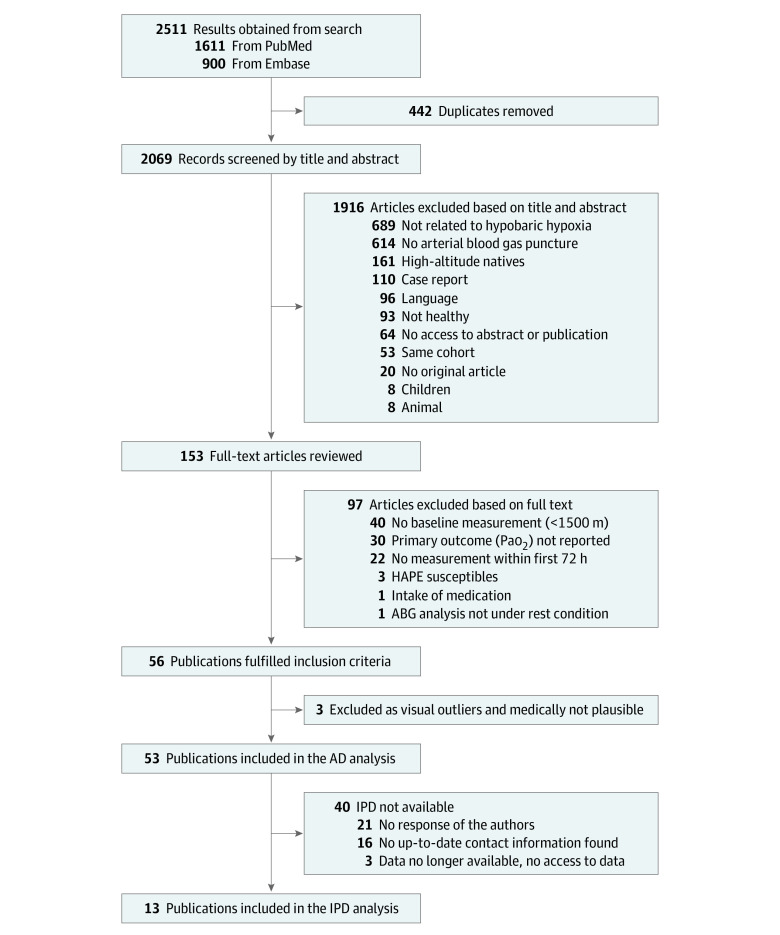
Study Selection Flowchart ABG indicates arterial blood gas; AD, aggregated data; HAPE, high-altitude pulmonary edema; and IPD, individual participant data.

The study selection flowchart is shown in Figure 1. The literature search provided 2511 records. After exclusion of duplicates, 2069 records remained for screening. Of those, 153 full-text articles were reviewed, 97 of which were excluded. Overall, 56 original articles reporting data on ABGs under hypobaric hypoxic conditions among healthy volunteers met inclusion criteria. A Galbraith plot including those 56 studies^9,15,16,17,18,19,20,21,22,23,24,25,26,27,28,29,30,31,32,33,34,35,36,37,38,39,40,41,42,43,44,45,46,47,48,49,50,51,52,53,54,55,56,57,58,59,60,61,62,63,64,65,66,67,68,69^ identified 3 visual outliers,^67,68,69^ which were excluded (eResults and eFigure 1 in Supplement 1).

In total, 53 studies^9,15,16,17,18,19,20,21,22,23,24,25,26,27,28,29,30,31,32,33,34,35,36,37,38,39,40,41,42,43,44,45,46,47,48,49,50,51,52,53,54,55,56,57,58,59,60,61,62,63,64,65,66^ reporting 115 group ascents to altitudes between 1524 m and 8730 m were included in the aggregated data analysis. Two^49,59^ of those studies (3.8%) were not incorporated into the forest plot due to missing measurements of variance. For 13^54,55,56,57,58,59,60,61,62,63,64,65,66^ of those studies (24.5%) reporting 29 ascents, the IPD were retrieved and included in IPD analyses. All of the included studies^9,15,16,17,18,19,20,21,22,23,24,25,26,27,28,29,30,31,32,33,34,35,36,37,38,39,40,41,42,43,44,45,46,47,48,49,50,51,52,53,54,55,56,57,58,59,60,61,62,63,64,65,66^ were published between 1967 and 2022; characteristics of all included studies are shown in eTable 1 in Supplement 1. The funnel plot revealed an even distribution around the mean effect size; no reporting bias was detected (eFigure 2 in Supplement 1).

### Characteristics

The aggregated data analysis included all 777 participants, of whom 267 (34.4%) were women and 510 (65.6%) were men (Table). The mean (SD) age was 36.2 (10.5) years, and the mean (SD) BMI was 24.9 (2.1). The mean (SD) baseline ABG measurements were 12.2 (1.3) kPa for Pao_2_, 5.0 (0.4) kPa for Paco_2_, 96.7% (1.4%) for Sao_2_, and 7.41 (0.02) for pH. The mean baseline altitude was 444 m (range, 0-1400 m).

**Table.** Baseline Characteristics of Participants in Studies With Aggregated Data and Individual Participant Data

Characteristic	Value, mean (SD) [range]		*P* value
	Studies included in aggregated data analysis (N = 53)^a^	Studies included in individual participant data analysis (n = 13)	
Total participants, No.	777	305	NA
Sex, No. (%)			
Female	267 (34.4)	120 (39.3)	.42
Male	510 (65.6)	185 (60.7)	.12
Age, y	36.2 (10.5) [18.0-64.0]	39.8 (13.6) [18.0-67.2]	.30
Height, cm	176 (3) [164-192]	174 (9) [156-197]	.37
Weight, kg	73.2 (3.7) [55.1-112.3]	69.9 (12.4) [48.5-105.0)]	.10
BMI	24.9 (2.1) [21.8-27.8]	25.1 (3.8) [16.9-36.9]	.80
Baseline altitude, m	444 (389) [0-1400]	424 (364) [0-1400]	NA
Arterial blood gases at <1500 m			
pH	7.41 (0.02) [7.37-7.47]	7.41 (0.03) [7.35-7.52]	.99
Pao_2_, kPa	12.2 (1.3) [9.7-15.0]	11.9 (1.9) [8.2-19.9]	.50
Paco_2_, kPa	5.0 (0.4) [3.9-5.6]	5.2 (0.5) [3.1-6.2]	.13
Sao_2_, %	96.7 (1.4) [94.6-99.0]	96.1 (1.8) [90.8-100]	.20

Abbreviations: BMI, body mass index (calculated as weight in kilograms divided by height in meters squared); NA, not applicable; Sao_2_, arterial oxygen saturation.

^a^ For the aggregated data analysis, the mean was weighted by the number of participants included in each study.

The IPD analysis included 305 of 777 participants (39.3%), of whom 120 (39.3%) were women and 185 (60.7%) were men (Table). The mean (SD) age was 39.8 (13.6) years, and the mean (SD) BMI was 25.1 (3.8). For this subgroup, mean (SD) baseline ABG measurements were 11.9 (1.9) kPa for Pao_2_, 5.2 (0.5) kPa for Paco_2_, 96.1% (1.8%) for Sao_2_, and 7.41 (0.03) for pH. The mean baseline altitude was 424 m (range, 0-1400 m).

A total of 2418 ABG measurements were noted; of those, 891 were baseline measurements. Between the altitudes of 1500 m and 3000 m, 130 measurements were taken; between the altitudes of 3001 m and 5000 m, 1171 measurements were taken; and at altitudes higher than 5000 m, 226 measurements were taken. Barometric pressure was reported in 25 studies^17,21,22,23,24,25,26,30,32,33,34,36,39,40,41,44,45,49,50,53,58,59,61,64,65^ (47.2%).

### Meta-analysis

**Figure 2.**
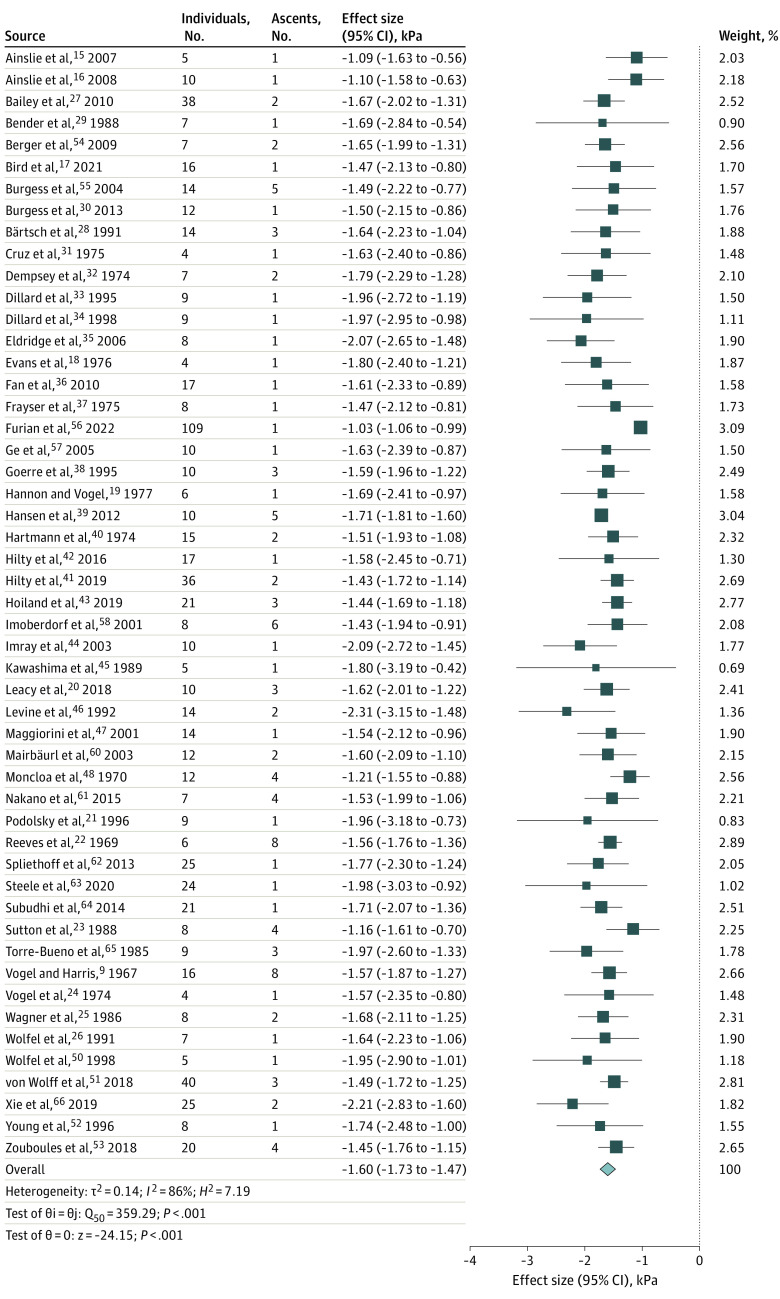
Changes in Pao_2_ by Altitude Based on Aggregated Data A total of 51 studies^9,15,16,17,18,19,20,21,22,23,24,25,26,27,28,29,30,31,32,33,34,35,36,37,38,39,40,41,42,43,44,45,46,47,48,50,51,52,53,54,55,56,57,58,60,61,62,63,64,65,66^ are shown in the final forest plot; 2 studies^49,59^ included in the aggregated data analysis are not shown because of incomplete data. All values necessary to determine the mean effect size (calculated as the ratio of the difference in Pao_2_ to the difference in altitude multiplied by 1000) for each study are provided in eTable 1 in Supplement 1. For example, Ainslie et al^15^ measured Pao_2_ at 1400 m and 3840 m, and found that the corresponding means of Pao_2_ were 9.92 kPa and 7.25 kPa, respectively. The mean effect size was therefore 1.09 kPa per 1000 m (9.92 kPa minus 7.25 kPa divided by 3840 m minus 1400 m then multiplied by 1000). Multilevel meta-analysis using a random-effects DerSimonian-Laird model was conducted to assess the pooled effect if a study had more than 1 measurement made at high altitude. The size of the squares corresponds to the weight of the effect size of the individual studies. The width of the diamond corresponds to the 95% CI of the point estimate of the pooled effect.

**Figure 3.**
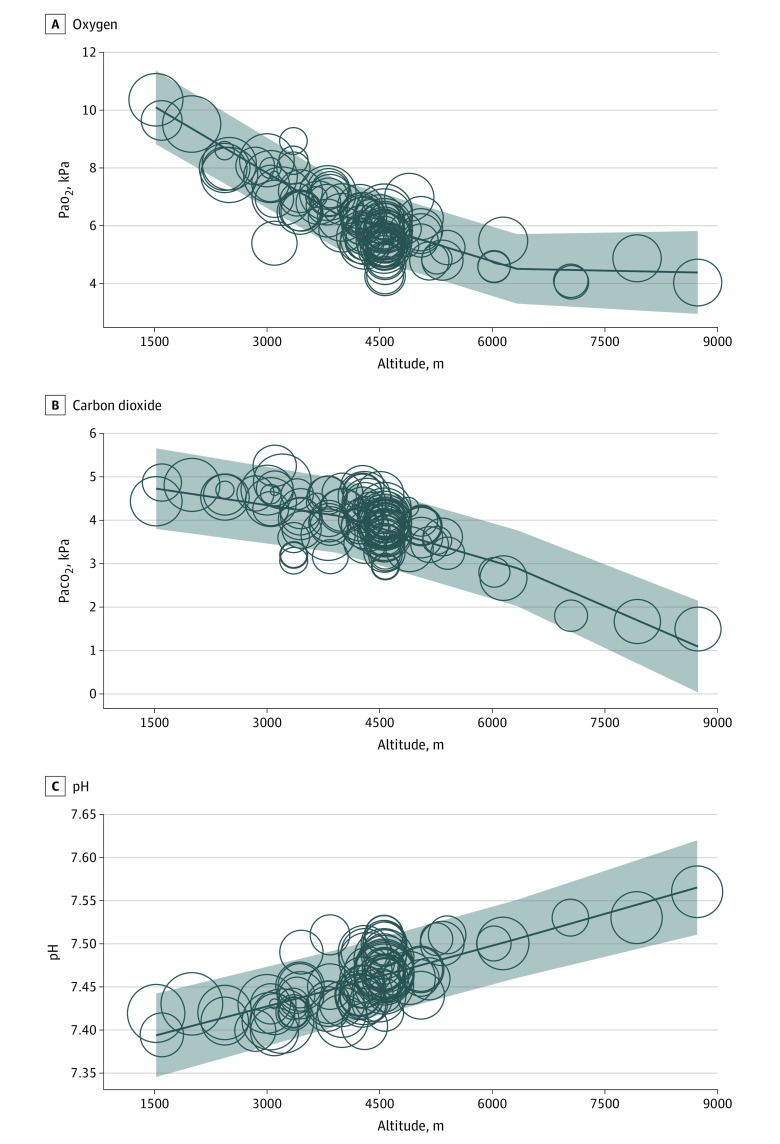
Arterial Blood Gas Values by Altitude Based on Aggregated Data The size of each bubble is proportional to the SE of each of the 51 studies^9,15,16,17,18,19,20,21,22,23,24,25,26,27,28,29,30,31,32,33,34,35,36,37,38,39,40,41,42,43,44,45,46,47,48,50,51,52,53,54,55,56,57,58,60,61,62,63,64,65,66^ shown in the figure; 2 studies^49,59^ included in the aggregated data analysis are not shown because of incomplete data. The 95% CI (gray area) is the SE of the estimate (ie, the SE of the point estimation for 1 observation).

The association of altitude (standardized for 1000 m vertical gain) with Pao_2_ is shown in the forest plot in Figure 2. The mean point estimate calculated for the 51 included studies^9,15,16,17,18,19,20,21,22,23,24,25,26,27,28,29,30,31,32,33,34,35,36,37,38,39,40,41,42,43,44,45,46,47,48,50,51,52,53,54,55,56,57,58,60,61,62,63,64,65,66^ was a reduction of 1.60 kPa in Pao_2_ per kilometer of vertical ascent. The 95% CI of the overall effect estimate was 1.47 to 1.73 kPa. The test for heterogeneity resulted in a τ^2^ value of 0.14, an *I*^2^ value of 86%, and an *H*^2^ value of 7.19 (test of θi = θj: *Q*_50_ = 359.29 [*P* < .001]; test of θ = 0: *z* = −24.15 [*P* < .001]). The bubble plot (Figure 3) revealed that most studies were conducted at an altitude of approximately 4500 m, and fewer studies were conducted at higher altitudes. Sensitivity analysis using log(Pao_2_) instead of raw Pao_2_ as a function of altitude revealed greater heterogeneity (*I*^2^ = 95%). Results from the exponential model for Pao_2_ and Paco_2_ obtained from the aggregated data analysis are shown in eFigure 3 in Supplement 1.

### Individual Participant Data

**Figure 4.**
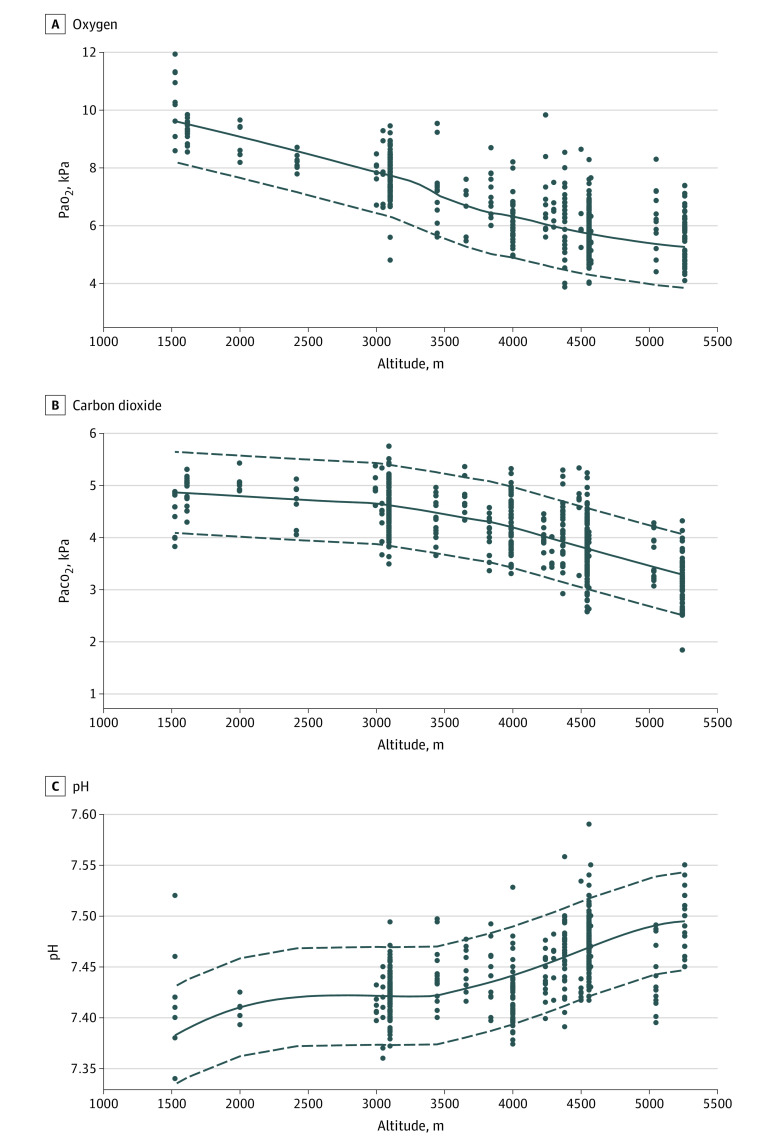
Lower and Upper Limits of Normal for Pao_2_, Paco_2_, and pH Based on Individual Participant Data A total of 13 studies^54,55,56,57,58,59,60,61,62,63,64,65,66^ were included in the analysis. Dots represent individual participant data, continuous lines represent means, and dashed lines represent 90% CIs. The lower dashed line represents the lower limit of normal and the upper dashed line represents the upper limit of normal at the respective altitude. The 90% CIs were not corrected for possible confounders, such as age, sex, and body mass index.

The distribution of the IPD and the overlaid 90% CI boundaries for Pao_2_, Paco_2_, and pH values in relation to altitude are shown in Figure 4. In the regression model for Pao_2_ at different altitudes, target altitude (−1.53 kPa per 1000 m; 95% CI, −1.63 to −1.42 kPa per 1000 m), age (−0.01 kPa per year; 95% CI, −0.02 to −0.003 kPa per year), and time spent at an altitude of 1500 m or higher, either during ascent or up to 3 days at the altitude of measurement (0.16 kPa per day; 95% CI, 0.11-0.21 kPa per day), were significantly associated with Pao_2_ (eTable 2 in Supplement 1). In this model, Pao_2_ decreased by 1.53 kPa with every 1000 m increase in altitude, which was consistent with our findings from the aggregated data analysis. Sex and Pao_2_ at LA were included in the model but were not significantly associated with Pao_2_. The corresponding equation to calculate Pao_2_ at HA was as follows:

Pao_2_ at HA = 13.185 − (1.525 × Target Altitude) − (0.013 × Age) + (0.122 × Sex) − (0.035 × Pao_2_ at LA) + (0.163 × Time at Altitude > 1500 m),

with Pao_2_ measured in kPa, target altitude in kilometers, age in years, time at altitude higher than 1500 m in days, and sex assigned a binary value (0 for male and 1 for female). Exploratory analysis using log(Pao_2_) as the dependent variable confirmed the results presented in eTable 2 in Supplement 1; however, age did not reach statistical significance (eTable 3 in Supplement 1).

### Risk of Bias Assessment

The risk of bias assessment was conducted for all 53 studies.^9,15,16,17,18,19,20,21,22,23,24,25,26,27,28,29,30,31,32,33,34,35,36,37,38,39,40,41,42,43,44,45,46,47,48,49,50,51,52,53,54,55,56,57,58,59,60,61,62,63,64,65,66^ No study was rated good, 46^9,17,18,19,20,22,23,24,25,26,27,28,29,30,31,32,33,34,35,36,37,38,39,40,41,42,43,44,45,46,47,48,49,50,51,52,53,54,55,56,60,61,62,63,64,66^ were rated fair, and 7^15,16,21,57,58,59,65^ were rated poor (eTable 4 in Supplement 1).

## Discussion

This systematic review and meta-analysis reported the results of ABG analyses conducted among healthy unacclimatized adult participants exposed to a hypobaric hypoxic environment. To our knowledge, no study has specifically investigated changes in ABG values in a larger population exposed to a similarly wide range of altitudes to derive effect size estimates. We found that for 1000 m of vertical ascent, Pao_2_ decreased by 1.60 kPa (95% CI, 1.47-1.73 kPa). Furthermore, we provided an equation to estimate Pao_2_ at the target altitude based on baseline parameters. These novel insights into ABG parameters as a function of altitude may help to put the ABG measurements of altitude trekkers into context and improve understanding with regard to the clinical manifestations and physiological consequences of altitude. Moreover, as the accessibility of HA regions improves and more people travel to those regions, more chronically ill people will also visit HA locations. Our quantitative findings may improve preventive and therapeutic decision-making for these patients.

For our systematic review, we only considered studies measuring the acute consequences of HA for participants. In this study, acute was defined as less than 3 days (72 hours) of travel at the altitude of measurement to minimize the consequences of acclimatization. This definition of acute was chosen for practical reasons, representing a typical short (acute) trip to an HA region. However, acclimatization is a complex process, starting from the moment of exposure and persisting for several weeks.^3^ The process of acclimatization to HA depends on various factors and is beyond the scope of this review. With the focus only on acute altitude outcomes, we created a more homogeneous pool of measurements. Studies of individuals at very high altitudes (VHAs) or extreme altitudes (EAs) with predefined ascent protocols implemented among all participants (and therefore a degree of acclimatization) were included because it was not realistic that VHA would be reached without any form of acclimatization. However, our definition of acute still required that no more than 3 days be spent at the altitude of measurement.

Another source of heterogeneity in altitude-related measurements might arise from the fact that altitude does not translate to the same Pb and therefore the same PiO_2_ across the globe. At the equator (0° latitude), Pb is higher than for a corresponding altitude at latitudes near the poles (−90° or 90° latitude), which leads to different Pb at the same altitude. Using Pb in a model has several benefits compared with altitude, with 1 benefit being the linear ratio of Pb to Po_2_ in the ambient air, representing the decrease in PiO_2_ and therefore the reduction in Pao_2_ more directly. Furthermore, if only altitude is used in a model, information about different Pb at the same altitude but different latitude is lost. However, Pb has several disadvantages that limit its general usability and made it ineligible for our purposes. It can only be measured with a barometer on site and, more important, it is dependent on weather conditions; in extreme cases, Pb can fluctuate up to 22 mm Hg, equivalent to 228 m. If Pb is not measured during a specific study period, it is no longer accessible because, to our knowledge, no map includes information about Pb in relation to latitude and altitude. The dependence on the availability of a reliable barometer and not just a map possibly leads to less reporting of Pb in studies; in all 53 included studies,^9,15,16,17,18,19,20,21,22,23,24,25,26,27,28,29,30,31,32,33,34,35,36,37,38,39,40,41,42,43,44,45,46,47,48,49,50,51,52,53,54,55,56,57,58,59,60,61,62,63,64,65,66^ only 25^17,21,22,23,24,25,26,30,32,33,34,36,39,40,41,44,45,49,50,53,58,59,61,64,65^ (47.2%) reported Pb. As mentioned, the use of Pb instead of altitude was not superior in explaining the variance in Pao_2_ at altitude.

On the level of an individual human’s reaction to hypoxemia, the hypoxic ventilatory response (HVR), which describes the natural response of hyperventilation in humans, plays an important role. When Pao_2_ decreases, HVR is different for every individual, and the different HVR result occurs in response to a given hypoxic stimulus and altitude. Moreover, hyperventilation-induced alkalemia and left shift of the oxyhemoglobin dissociation curve directly alter Sao_2_ and other ABG values. Therefore, it has been suggested that HVR may be associated with individual tolerance of VHA.^70,71^ Thus, it is harder to estimate the Pao_2_ for an individual person. However, because we reported a large number of measurements, we likely covered a broad range of HVRs, and very high and low individual HVRs did not change the robustness of the mean values.

Notably, in Figure 3, the curve of Pao_2_ seemed to plateau toward EAs. Whether this asymptotic behavior corresponds to a physiological barrier of minimally tolerable Pao_2_ remains to be elucidated. Every individual reaches an altitude at which they cannot tolerate hypoxemia further; for some, this limit might be at lower altitudes. For an individual, the Pao_2_ curve might decrease until the maximal tolerable altitude is reached, but the mean value of Pao_2_ might remain stable for a range of altitudes because mountaineers reach their plateaus at different altitudes. The nonlinearity might also be partially due to acclimatization because mountaineers ascending to EAs likely spent a substantial amount of time at HA before reaching these EAs.

A graphical overview of the IPD and range of values is provided in Figure 4. We chose the 90% CI to show the LLN and ULN. Panels A to C in Figure 4 are, to our knowledge, some of the first to describe normal values in a large number of individuals at different altitudes, creating the possibility to compare personal measurements or gauge whether values were within the expected range. However, the calculated 90% CI might overestimate the LLN for older people and underestimate it in young athletic people. Nevertheless, those figures (Figure 4A-C) are of clinical value to determine a range for Pao_2_ at various altitudes in men and women of any age and weight.

For a more exact estimate of the values that can be expected for individuals when traveling to mountainous regions, we created an estimation model for Pao_2_ using baseline measurements. To apply this model, only basic demographic information and a baseline ABG are required. As expected, the target altitude was significantly associated with the expected Pao_2_. Younger age and more time spent at an altitude of 1500 m or higher (either during ascent or up to 3 days at the target altitude) were significantly associated with higher Pao_2_ values at the target altitude. These factors are already known to be associated with Pao_2_^72^; moreover, a slow ascent to HA is recommended to minimize the risk of AMS and allow the body to acclimatize.^71^ The finding that baseline Pao_2_ was not associated with Pao_2_ at HA was notable because 1 study^73^ reported altitude-related changes in ABG at altitudes lower than 1500 m. One possible explanation is that other confounding factors play a more substantial role in Pao_2_ at HA. This hypothesis was confirmed in the exploratory analysis using log(Pao_2_) as the dependent variable (eTable 3 in Supplement 1).

### Limitations

This study has several limitations. One of the challenges faced was the quantitatively and qualitatively heterogeneous composition of participants over different altitudes. After excluding 3 outlier studies,^67,68,69^ the percentage of total variability remained high (*I*^2^ = 86%). Further approaches for lowering heterogeneity by using an exponential model of Pao_2_ and altitude or Pb were unsuccessful. Especially when using VHA measurements, a certain bias is unavoidable because only people with the physical capacity as well as the physiological and psychological preconditions to reach those altitudes can be tested, and acclimatization would have occurred. Therefore, at VHA, our data do not represent the general population, and the gap between measurements in the general population and those obtained in well-trained mountaineers will increase with altitude.

## Conclusions

This systematic review and meta-analysis provided estimates of reductions in Pao_2_ among healthy adults at altitudes of 1500 m or higher. With every kilometer of vertical gain, Pao_2_ decreased by a mean of 1.60 kPa. This study also provides reference values for the general population and an approximation of the LLN for Pao_2_ at altitudes ranging from 1500 m to 5260 m. These reference values and estimation equations may enhance understanding of altitude-related physiological mechanisms and support clinical decision-making when altitude-related adverse health effects occur in healthy individuals or in patients with preexisting diseases who are traveling to HA regions.

## Notes

Supplement 1. **eMethods.** Literature Search, Outcomes, Study Risk of Bias Assessment, and Statistical Analysis **eResults.** Study Exclusion and Risk of Bias Assessment **eTable 1.** Characteristics of Included Studies **eTable 2.** Baseline Factors Associated With Pao_2_ at Altitude Based on Individual Participant Data **eTable 3.** Exploratory Regression Analysis of Individual Participant Data Using Log Pao_2_ as Dependent Variable **eTable 4.** Risk of Bias Assessment **eFigure 1.** Galbraith Plot **eFigure 2.** Funnel Plot **eFigure 3.** Arterial Blood Gas Values Expressed by Percentage From Low Altitude
eReferences

Supplement 2.
Data Sharing Statement
