# Supplementary material for: Partial Pressure of Arterial Oxygen in Healthy Adults at High Altitudes: A Systematic Review and Meta-Analysis
Source: JAMA Netw Open. 2023 Jun 16;6(6):e2318036. doi: 10.1001/jamanetworkopen.2023.18036 (PMC10276310; doi:10.1001/jamanetworkopen.2023.18036)
Supplement: Supplement 1. — eMethods. Literature Search, Outcomes, Study Risk of Bias Assessment, and Statistical Analysis eResults. Study Exclusion and Risk of Bias Assessment eTable 1. Characteristics of Included Studies eTable 2. Baseline Factors Associated With Pao2 at Altitude Based on Individual Participant Data eTable 3. Exploratory Regression Analysis of Individual Participant Data Using Log Pao2 as Dependent Variable eTable 4. Risk of Bias Assessment eFigure 1. Galbraith Plot eFigure 2. Funnel Plot eFigure 3. Arterial Blood Gas Values Expressed by Percentage From Low Altitude eReferences [file jamanetwopen-e2318036-s001.pdf]

Supplementary Online Content

Forrer A, Gaisl T, Sevik A, et al. Partial pressure of arterial oxygen in healthy adults at high altitudes: a systematic review and meta-analysis. *JAMA Netw Open*. 2023;6(6):e2318036. doi:10.1001/jamanetworkopen.2023.18036

eMethods. Literature Search, Outcomes, Study Risk of Bias Assessment, and Statistical Analysis

eResults. Study Exclusion and Risk of Bias Assessment

eTable 1. Characteristics of Included Studies

eTable 2. Baseline Factors Associated With Pa_{O_2} at Altitude Based on Individual Participant Data

eTable 3. Exploratory Regression Analysis of Individual Participant Data Using Log Pa_{O_2} as Dependent Variable

eTable 4. Risk of Bias Assessment

eFigure 1. Galbraith Plot

eFigure 2. Funnel Plot

eFigure 3. Arterial Blood Gas Values Expressed by Percentage From Low Altitude

eReferences

This supplemental material has been provided by the authors to give readers additional information about their work.

© 2023 Forrer A et al. *JAMA Network Open*.

eMethods. Literature Search, Outcomes, Study Risk of Bias Assessment, and Statistical Analysis

Literature search

Databases of PubMed (Medline, PubMed Central, Bookshelf) and Embase were systematically searched for literature with the 11.04.2023 as cut-off date. Search terms employed for identifying eligible studies were:

In PubMed: (blood OR (arterial blood)) AND (gas OR gases) AND (altitude OR (hypobaric chamber) OR (altitude simulation) OR flight) NOT (review [Publication Type])

+ filter: Human and > 19 (age)

In Embase: (blood OR 'arterial blood') AND (gas OR gases) AND (altitude OR 'hypobaric chamber' OR 'altitude simulation' OR flight).

+ filter: Human and 18-64years, 65+ and 80+ years

Titles and Abstracts of the obtained results were screened and if they met the inclusion criteria, the full texts were obtained.

Outcomes

Furthermore, basic technical information about how the ABG was performed and which device was used were assembled. The same set of information was extracted if IPD was available. In addition, the ascent protocol and funding sources were noted for all studies included. Missing secondary outcomes were not replaced, no extrapolations or assumptions were made.

Study risk of bias assessment

Two independent researchers assessed the risk of bias using the Quality Assessment Tool for Observational Cohort and Cross-Sectional Studies (National Heart, Lung, and Blood Institute at the National Institutes of Health, USA).[12] The tool is composed of 14 individual questions, each answered with “yes”, “no” or “NR” (not reported) if no answer could be found within the publication. The studies were classified according to the system used by Bagias et al., 2021:[13] Studies with <4 of 14 questions answered with “yes” were rated as “poor – high risk of bias”, studies with 5-10 of 14 questions answered with “yes” were rated as “fair – moderate risk of bias” and studies with 11-14 of 14 questions answered with “yes” were rated as “good – low risk of bias”. Two questions of the applied tool were particularly important for this meta-analysis since they directly influence the PaO_2 variability: Question number 9 “Were the exposure measures (exposure time to hypobaric

© 2023 Forrer A et al. *JAMA Network Open*.

hypoxia) clearly defined, valid, reliable, and implemented consistently across all study participants?" and question number 11 "Were the outcome measures (ABG procedure) clearly defined, valid, reliable, and implemented consistently across all study participants?". In accordance, studies were rated as "poor" if either one of these two questions were negated.

Furthermore, in three studies, measurements were performed at different altitudes or after different times at the same high altitude location. In these studies, question number 13 ("Was loss to follow-up after baseline 20% or less?") was answered for each situation separately.

Statistical analysis

For outlier detection a Galbraith plot as well as physiological plausibility (as discussed among the team) served as a basis for the decision of study exclusion. A funnel plot was created to check for publication bias. Regression models including latitude or barometric pressure of the target altitude as predictors for PaO_2 were not superior to a linear regression model (data not shown). Therefore, outcomes were analyzed using linear regression models adjusted for altitude. The a-priori linearity assumption was tested for independence, linearity, homoscedasticity of the residuals and normality of the residuals. Then, the data was converted to the altitude effect (for mean effect: difference in kPa/difference in meters*1000; for mean standard error [SE]:). Because a subgroup of studies (n=19) published data on multiple assessments (range from 2 to 8) in the same individuals, a fixed-effects intra-study multivariate meta-analysis (using the methods described in section 16.1.3.2 of the Cochrane handbook)[14] was performed to account for the dependence among the outcomes. There was no evidence for significant heterogeneity between assessments (global p-value 0.681); and the calculated average estimate of the correlation between ascents was r=0.53. Two studies[15,16] presenting only the mean PaO_2 without variance were excluded from the overall estimate calculated by the forest plot. The calculated overall estimate of each study was then incorporated in an inter-study DerSimonian–Laird model random-effects multivariate meta-analysis. Heterogeneity was assessed using the estimated between-study variance (τ^2), and the I^2 statistic. The prediction model for PaO_2 at different altitudes was developed by a stepwise forward approach using mixed regression analysis and based on baseline characteristics of IPD. Predictors were the target altitude, age, gender, BMI, PaO_2 at low altitude (<1500 m) and time spent above 1500 m. Improvement of the model was determined with the Akaike and Bayesian Information Criterion. To determine predictors in the final model a p-value <0.05 was considered statistically significant. Due to the

potential exponential relationship between PaO_2 and altitude, sensitivity analyses were performed using log (PaO_2) as dependent variable. In the IPD, the lower 5% confidence interval boundary was calculated for PaO_2, $PaCO_2$ and pH and represents the lower limit of normal (LLN) as a function of altitude. Correspondingly, the upper 95% confidence interval boundary was calculated for $PaCO_2$ and pH since values above the upper limit of normal (ULN) do represent abnormal adaptations to altitude. All statistical analyses were performed in STATA v17 and specifically included the family of commands for meta-analysis.

© 2023 Forrer A et al. *JAMA Network Open*.

eResults. Study Exclusion and Risk of Bias Assessment

A Galbraith plot including those 56 studies showed three visual outliers, which we excluded based on this plot (**eFigure 1, eResults).** In the study of Yang et al.[17] $PaCO_2$ rose from baseline to 3568 m and PaO_2 hardly changed. Since no explanation was provided by the authors and based on the Galbraith plot the study was excluded. The other two excluded studies[18,19] showed similar physiologically unrealistic results and lay far out on the Galbraith plot.

Risk of bias assessment

The risk of bias assessment was done for all 53 studies. No study was rated "good", 47 were rated "fair," and 7 were rated "poor" (Supplement eTable 5). As the ABGs were almost never the primary outcome and blinding both the participants and the outcome assessors to the exposure status (here: high altitude) was logistically challenging to implement, question No. 12 and 14 were always answered negative. Often no flowchart presenting the inclusion process was displayed; therefore, question 3 (“was the participation rate of eligible persons at least 50%?") was usually scored as NR.

© 2023 Forrer A et al. *JAMA Network Open*.

eTable 1. Characteristics of Included Studies

Index	Publication	Altitude [m]	Analyzer	N, % male	Demographics	Arterial blood gases at BL [kPa]	Arterial blood gases at HA [kPa]	ARAHE, overall
1	Kann et al.[1], 1967	BL: 0 / 460<br>HA: 1614 / 2418[2]	NR	23, NR%	Age, y: range 19-40;<br>BMI, kg/m^2: NR	PaO_2, kPa: 13.05 / 11.45<br>$PaCO_2$, kPa: 5.0 / 4.91<br>SaO_2, %: NR<br>pH: NR	PaO_2, kPa: 9.29 / 8.2<br>$PaCO_2$, kPa: 4.91/ 4.68<br>SaO_2, %: NR<br>pH: NR	NR
2	Reeves et al.[2], 1967	BL: 304.8<br>HA: 3108.96[2]	radiometer electrodes in Leadville (HA) and Instrumentation Laboratory system in Lexington (SL)	1, 100%	Age, y: 35;<br>BMI, kg/m^2: 22.7	PaO_2, kPa: 13.07<br>$PaCO_2$, kPa: NR<br>SaO_2, %: 97<br>pH: 7.41	PaO_2, kPa: 7.60<br>$PaCO_2$, kPa: NR<br>SaO_2, %: 88<br>pH: 7.4	0
3	Vogel et al.[3], 1967	BL: 0<br>HA: 3352.8[1] / 4572[1]	Radiometer (PaO_2, $PaCO_2$ and pH), American Optical (reflection oximeter) for O_2 saturation	16, 100%	Age, y: range 19-26;<br>BMI, kg/m^2: NR	PaO_2, kPa: 13.33±0.95<br>$PaCO_2$, kPa: 4.00±0.27<br>SaO_2, %: 99±1<br>pH: 7.39±0.01	PaO_2, kPa: 8.00±0.67 / 6.00±0.4<br>$PaCO_2$, kPa: 3.6±0.4 / 2.93±0.27<br>SaO_2, %: 93±2 / 83 ± 4<br>pH: 7.43±0.03 / 7.45±0.02	NR
4	Reeves et al.[4], 1969	BL: 0<br>HA: 4572[1]	Model 113, Instrumentation Laboratory	9, 100%	Age, y: 26 range 21-36;<br>BMI, kg/m^2: NR	PaO_2, kPa: 11.6±0.67<br>$PaCO_2$, kPa: 4.8±0.27<br>SaO_2, %: NR<br>pH: 7.41±0.02	PaO_2, kPa: 5.2±0.53<br>$PaCO_2$, kPa: 4.27±0.27<br>SaO_2, %: NR<br>pH: 7.46±0.02	NR
5	Moncloa et al.[5], 1970	BL: 0<br>HA: 4267[2]	Model 27, Radiometer, Clark electrode and Severinghaus electrode	12, 100%	Age, y: range 19-29;<br>BMI, kg/m^2: NR	PaO_2, kPa: 11.33±1.0<br>$PaCO_2$, kPa: 5.43±0.28<br>SaO_2, %: NR<br>pH: 7.417±0.03	PaO_2, kPa: 6.4±0.94<br>$PaCO_2$, kPa: 4.79±0.38<br>SaO_2, %: NR<br>pH: 7.43±0.0.03	NR
6	Dempsey et al.[6], 1974	BL: 250[1,2]<br>HA: 3100[1,2]	Radiometer	7, 100%	Age, y: range 24-44;<br>BMI, kg/m^2: NR	PaO_2, kPa: 11.47±0.66<br>$PaCO_2$, kPa: 5.4±0.29<br>SaO_2, %: NR<br>pH: 7.391±0.011	PaO_2, kPa: 5.4±0.82 / 8.04±0.24<br>$PaCO_2$, kPa: 5.25±0.32 / 4.29±0.21<br>SaO_2, %: NR	0

© 2023 Forrer A et al. *JAMA Network Open*.

Index	Publication	Altitude [m]	Analyzer	N, % male	Demographics	Arterial blood gases at BL [kPa]	Arterial blood gases at HA [kPa]	ARAHE, overall
							pH: 7.398±0.013 / 7.421±0.013	
7	Hartmann et al.[7], 1974	BL: 524[2]<br>HA: 1600 / 2844[2]	Gas-Check-AVL-Mikroblutgasanalysator	15, 26.7%	Age, y: 33.4 range 21-61;<br>BMI, kg/m^2: NR	PaO_2, kPa: 11.37±0.99<br>$PaCO_2$, kPa: 5.29±0.37<br>SaO_2, %: NR<br>pH: 7.369±0.021	PaO_2, kPa: 9.64±0.78 / 8.07±0.55<br>$PaCO_2$, kPa: 4.87±0.47 / 4.62±0.4<br>SaO_2, %: NR<br>pH: 7.394±0.025 / 7.399±0.024	NR
8	Vogel et al.[8], 1974	BL: 756[2]<br>HA: 4350[2]	NR	4, 100%	Age, y: 21±0.82 range 20-22;<br>BMI, kg/m^2: 23.45±1.64	PaO_2, kPa: 12.39±0.7<br>$PaCO_2$, kPa: NR<br>SaO_2, %: 96.7±4.8<br>pH: NR	PaO_2, kPa: 5.53±0.38<br>$PaCO_2$, kPa: NR<br>SaO_2, %: 80.6±3.8<br>pH: NR	NR
9	Cruz et al.[9], 1975	BL: 150[2]<br>HA: 4350[2]	Van Slyke procedure, Instrumentation Laboratory	4, 100%	Age, y: 21±0.8 range 20-22;<br>BMI, kg/m^2: NR	PaO_2, kPa: 12.39±0.7<br>$PaCO_2$, kPa: 5.24±0.62<br>SaO_2, %: NR<br>pH: 7.413±0.066	PaO_2, kPa: 5.53±0.38<br>$PaCO_2$, kPa: 3.76±0.3<br>SaO_2, %: NR<br>pH: 7.468±0.088	NR
10	Frayser et al.[10], 1975	BL: 1[2]<br>HA: 2926 / 5334[2]	Instrumentation Laboratory	8, 100%	Age, y: range 19-37;<br>BMI, kg/m^2: NR	PaO_2, kPa: 11.52±0.45<br>$PaCO_2$, kPa: 4.81±0.42<br>SaO_2, %: 98±2.8<br>pH: 7.47±0.03	PaO_2, kPa: 7.6±0.14 / 4.81±0.82<br>$PaCO_2$, kPa: 3.93±0.31 / 3.61±0.25<br>SaO_2, %: NR<br>pH: 7.48±0.03 / 7.51±0.03	NR
11	Evans et al.[11], 1976	BL: 200[2]<br>HA: 4300[2]	NR	4, NR	Age, y: range 18-27;<br>BMI, kg/m^2: NR	PaO_2, kPa: 13.15±0.54<br>$PaCO_2$, kPa: 5.25±0.42<br>SaO_2, %: NR<br>pH: 7.401±0.026	PaO_2, kPa: 5.75±0.3<br>$PaCO_2$, kPa: 4.13±0.42<br>SaO_2, %: NR<br>pH: 7.45±0.02	NR
12	Hannon et al.[12], 1977	BL: 200[2]<br>HA: 4300[2]	Radiometer	6,100%	Age, y: range 20-23;<br>BMI, kg/m^2: NR	PaO_2, kPa: 12.67±0.8<br>$PaCO_2$, kPa: 5.4±0.38<br>SaO_2, %: 97<br>pH: 7.4±0.017	PaO_2, kPa: 5.73±0.42<br>$PaCO_2$, kPa: 4.41±0.29<br>SaO_2, %: 77<br>pH: 7.404±0.017	NR

© 2023 Forrer A et al. *JAMA Network Open*.

Index	Publication	Altitude [m]	Analyzer	N, % male	Demographics	Arterial blood gases at BL [kPa]	Arterial blood gases at HA [kPa]	ARAHE, overall
13	Torre-Bueno et al.[13], 1985	BL: 0[1]<br>HA: 1524 / 3048 / 4572[1]	NR	9, 100%	Age, y: 26±6 range 19-35;<br>BMI, kg/m^2: 22.94±1.8	PaO_2, kPa: 13.7±1.35<br>$PaCO_2$, kPa: 4.57±0.65<br>SaO_2, %: NR<br>pH: 7.439±0.046	PaO_2, kPa: 10.36±1.05 / 7.7±0.95 / 5.65±0.9<br>$PaCO_2$, kPa: 4.44±0.38 / 4.42±0.47 / 3.71±0.44<br>SaO_2, %: NR<br>pH: 7.419±0.047 / 7.41±0.03 / 7.48±0.35	NR
14	Wagner et al.[14], 1986	BL: 0[1]<br>HA: 3048 / 4572[1]	IL 213 blood gas electrode system, Instrumentation Laboratory	8, 87.5%	Age, y: 29.75±5.74 range 25-41;<br>BMI, kg/m^2: 22.3±1.9	PaO_2, kPa: 12.67±0.67<br>$PaCO_2$, kPa: 4.53±0.27<br>SaO_2, %: NR<br>pH: 7.42±0.02	PaO_2, kPa: 7.47±0.4 / 5.2±0.8<br>$PaCO_2$, kPa: 4.27±0.53 / 4.0±0.53<br>SaO_2, %: NR<br>pH: 7.43±0.03 / 7.46±0.02	NR
15	Bender et al.[15], 1988	BL: 0[1]<br>HA: 4292.25[1] / 4300[2]	ABL 300, Radiometer	7, 100%	Age, y: 22±1;<br>BMI, kg/m^2: NR	PaO_2, kPa: 12.86±1.46<br>$PaCO_2$, kPa: 4.93±0.34<br>SaO_2, %: 96±1.06<br>pH: 7.4±0.026	PaO_2, kPa: 5.61±0.56 / 6.66±0.37<br>$PaCO_2$, kPa: 4.55±0.29 / 3.33±0.29<br>SaO_2, %: 81±5.29 / 86±2.65<br>pH: 7.44±0.026 / 7.46±0.026	NR
16	Sutton et al.[16], 1988	BL: 0[1]<br>HA: 4093.18 / 6143.05 / 7923.22 / 8730.02[1]	ABL 3, Radiometer, IL 282 CO-Oximeter, Instrumentation Laboratory	8, 100%	Age, y: range 21-31;<br>BMI, kg/m^2: NR	PaO_2, kPa: 13.24±1.24<br>$PaCO_2$, kPa: 4.52±0.47<br>SaO_2, %: 97.6±0.1<br>pH: 7.43±0.04	PaO_2, kPa: 6.99±0.53 / 5.48±0.44 / 4.88±0.29 / 4.04±0.28<br>$PaCO_2$, kPa: 3.33±0.29 / 2.67±0.37 / 1.67±0.15 / 1.49±0.23<br>SaO_2, %: 84.8±4 / 75.2±6 / 67.8±5 / 58±4.5<br>pH: 7.46±0.02 / 7.5±0.04 / 7.53±0.03 / 7.56±0.03	NR

© 2023 Forrer A et al. *JAMA Network Open*.

Index	Publication	Altitude [m]	Analyzer	N, % male	Demographics	Arterial blood gases at BL [kPa]	Arterial blood gases at HA [kPa]	ARAHE, overall
17	Kawashima et al.[17], 1989	BL: 0[1]<br>HA: 3200[1]	ABL 2, Radiometer	5, 100%	Age, y: 24.4±0.5 range 23-26;<br>BMI, kg/m^2: NR	PaO_2, kPa: 11.69±1.54<br>$PaCO_2$, kPa: 5.16±0.45<br>SaO_2, %: NR<br>pH: 7.39±0.02	PaO_2, kPa: 7.03±0.29<br>$PaCO_2$, kPa: 4.89±0.29<br>SaO_2, %: NR<br>pH: 7.41±0.02	NR
18	Bärtsch et al.[18], 1991	BL: 490[2]<br>HA: 4559[2]	278 blood gas system, Ciba Corning Diagnostics	7, 100%	Age, y: 30 range 20-64;<br>BMI, kg/m^2: NR	PaO_2, kPa: 12.29±1.27<br>$PaCO_2$, kPa: 5.21±0.5<br>SaO_2, %: 96±2.65<br>pH: NR	PaO_2, kPa: 5.24±0.63<br>$PaCO_2$, kPa: 4.12±0.21<br>SaO_2, %: 70±6.88<br>pH: NR	Yes, but not how many…
19	Wolfel et al.[19], 1991	BL: 0[2]<br>HA: 4300[2]	ABL 300, Radiometer	7, 100%	Age, y: 23±5.3;<br>BMI, kg/m^2: NR	PaO_2, kPa: 12.53±0.71<br>$PaCO_2$, kPa: 5.33±0.35<br>SaO_2, %: 97±2.65<br>pH: NR	PaO_2, kPa: 5.47±0.35<br>$PaCO_2$, kPa: 4.0±0.35<br>SaO_2, %: 81±2.65<br>pH: NR	NR
20	Levine et al.[20], 1992	BL: 0[1]<br>HA: 2500[1]	ABL 4, OSM 3 hemoximeter, Radiometer	Two groups: 7 / 14	Age, y: 26±8 / 27±7;<br>BMI, kg/m^2: NR	PaO_2, kPa: 14.96±0.83 / 13.76±0.55<br>$PaCO_2$, kPa: NR<br>SaO_2, %: 98.3±0.3 / 97.8±0.2<br>pH: NR	PaO_2, kPa: 7.76±0.32 / 8.15±0.17<br>$PaCO_2$, kPa: NR<br>SaO_2, %: 91.1±1.3 / 92.3±0.6<br>pH: NR	NR
21	Dillard et al.[21], 1995	BL: 0[1]<br>HA: 2438[1]	model 1312, Instrumentation Laboratory	9, 100%	Age, y: 33±5;<br>BMI, kg/m^2: NR	PaO_2, kPa: 12.75±1.03<br>$PaCO_2$, kPa: 5.25±0.53<br>SaO_2, %: NR<br>pH: 7.41±0.01	PaO_2, kPa: 7.97±0.56<br>$PaCO_2$, kPa: 4.59±0.53<br>SaO_2, %: NR<br>pH: 7.41±0.02	NR
22	Goerre et al.[22], 1995	BL: 490[2]<br>HA: 4559[2]	278 blood gas system, Ciba Corning Diagnostics	10, 63.64%	Age, y: range 21-54;<br>BMI, kg/m^2: NR	PaO_2, kPa: 12.33±0.77<br>$PaCO_2$, kPa: 5.12±0.52<br>SaO_2, %: NR<br>pH: 7.42±0.03	PaO_2, kPa: 5.81±0.61<br>$PaCO_2$, kPa: 3.8±0.31<br>SaO_2, %: NR<br>pH: 7.5±0.03	11
23	Podolsky et al.[23], 1996	BL: 0[2]<br>HA: 3810[2]	IL 813 blood gas analyzer and IL 282 CO-oximeter, Instrumentation Laboratory	9, 100%	Age, y: 29.4±5.2 range 25-39;<br>BMI, kg/m^2: 24.12±1.5	PaO_2, kPa: 14.59±1.49<br>$PaCO_2$, kPa: 4.4±0.52<br>SaO_2, %: NR<br>pH: NR	PaO_2, kPa: 7.13±1.13<br>$PaCO_2$, kPa: 3.67±0.67<br>SaO_2, %: NR<br>pH: NR	0

© 2023 Forrer A et al. *JAMA Network Open*.

Index	Publication	Altitude [m]	Analyzer	N, % male	Demographics	Arterial blood gases at BL [kPa]	Arterial blood gases at HA [kPa]	ARAHE, overall
24	Young et al.[24], 1996	BL: 0[2]<br>HA: 4300[2]	ABL 300, OSM 3 hemoximeter, Radiometer	8, 100%	Age, y: 31±2.8;<br>BMI, kg/m^2: NR	PaO_2, kPa: 13.73±0.76<br>$PaCO_2$, kPa: 4.8±0.76<br>SaO_2, %: 96.8<br>pH: 7.42±0.028	PaO_2, kPa: 6.27±0.76<br>$PaCO_2$, kPa: 3.47±0.37<br>SaO_2, %: 83.2<br>pH: 7.49±0.028	NR
25	Dillard et al.[25], 1998	BL: 0[1]<br>HA: 2438[1]	NR	9, 100%	Age, y: 33±5;<br>BMI, kg/m^2: NR	PaO_2, kPa: 12.8±1.07<br>$PaCO_2$, kPa: 5.2±0.53<br>SaO_2, %: NR<br>pH: 7.4±0.02	PaO_2, kPa: 8.0±1.07<br>$PaCO_2$, kPa: 4.53±0.53<br>SaO_2, %: NR<br>pH: 7.42±0.02	NR
26	Wolfel et al.[26], 1998	BL: 0[2]<br>HA: 4300[2]	ABL 300, Radiometer	5, 100%	Age, y: 26.7±3.98;<br>BMI, kg/m^2: NR	PaO_2, kPa: 13.73±0.89<br>$PaCO_2$, kPa: 5.33±0.29<br>SaO_2, %: 97±2.24<br>pH: 7.4±0.022	PaO_2, kPa: 5.33±0.6<br>$PaCO_2$, kPa: 3.87±0.29<br>SaO_2, %: 79±4.47<br>pH: 7.49±0.022	NR
27	Imoberdorf et al.[27], 2001	BL: 550[2]<br>HA: 4559[2]	NR	Two groups<br>8, 62.5%<br>9, 66.7%	Age, y: 32.6±9.1 range 24-53 / 37.8±8.9 range 28-52;<br>BMI, kg/m^2: 23.3±2.1 / 23.6±4.4	PaO_2, kPa: 12.83±1.67 / 11.83±1.83<br>$PaCO_2$, kPa: 5.13±0.33 / 5.28±0.42<br>SaO_2, %: 96.7 / 95.3<br>pH: 7.435±0.022 / 7.43±0.019	PaO_2, kPa: 6.33±0.58 / 5.5±0.5<br>$PaCO_2$, kPa: 4.0±0.52 / 4.28±0.58<br>SaO_2, %: 80.7±4 / 73.3±7.7<br>pH: 7.468±0.018 / 7.453±0.02	2 / 3
28	Maggiorini et al.[28], 2001	BL: 490[2]<br>HA: 4559[2]	Model 287, Ciba-Corning Diagnostics	14, 76.7%	Age, y: range 23-60;<br>BMI, kg/m^2: NR	PaO_2, kPa: 12.27±1.01<br>$PaCO_2$, kPa: 4.53±0.49<br>SaO_2, %: NR<br>pH: NR	PaO_2, kPa: 6.0±0.49<br>$PaCO_2$, kPa: 3.6±0.49<br>SaO_2, %: NR<br>pH: NR	0
29	Imray et al.[29], 2003	BL: 150[2]<br>HA: 3459[2]	AVL OPTI 1 Blood Gas/pH Analyser, AVL List G.m.b.h.	10, 100%	Age, y: range 24-53;<br>BMI, kg/m^2: NR	PaO_2, kPa: 13.4±0.9<br>$PaCO_2$, kPa: 5.06±0.05<br>SaO_2, %: NR<br>pH: 7.4±0.03	PaO_2, kPa: 6.5±0.5<br>$PaCO_2$, kPa: 3.78±0.36<br>SaO_2, %: NR<br>pH: 7.49±0.02	0
30	Mairbäurl et al.[30], 2003	BL: 490[2]<br>HA: 4559[2]	model 287, Ciba-Corning Diagnostics	12, 66.7%	Age, y: 40.5±8.6 range 24-52;<br>BMI, kg/m^2: NR	PaO_2, kPa: 11.87±1.08<br>$PaCO_2$, kPa: 4.91±0.39<br>SaO_2, %: 94.9±6.1<br>pH: 7.413±0.026	PaO_2, kPa: 5.29±0.53<br>$PaCO_2$, kPa: 3.48±0.45<br>SaO_2, %: 77.1±5.8<br>pH: 7.485±0.026	NR

© 2023 Forrer A et al. *JAMA Network Open*.

Index	Publication	Altitude [m]	Analyzer	N, % male	Demographics	Arterial blood gases at BL [kPa]	Arterial blood gases at HA [kPa]	ARAHE, overall
31	Burgess et al.[31], 2004	BL: 0[2]<br>HA: 1400 / 3446 / 3840 / 4240 / 5050[2]	i-STAT, Abbott Diagnostics	14, 57.14%	Age, y: 35.8±9.8 range 20-52;<br>BMI, kg/m^2: 23.2±2.9	PaO_2, kPa: 12.67±0.93<br>$PaCO_2$, kPa: 5.6±0.4<br>SaO_2, %: NR<br>pH: 7.4±0.03	PaO_2, kPa: 10.35±0.8 / 7.2±1.07 / 7.07±0.93 / 6.67±1.2 / 6.27±1.07<br>$PaCO_2$, kPa: 5.2±0.4 / 4.27±0.4 / 3.87±0.53 / 4.0±0.27<br>SaO_2, %: NR<br>pH: 7.42±0.02 / 7.44±0.03 / 7.45±0.03 / 7.44±0.02 / 7.44±0.03	"almost all of the subjects suffered AMS on at least one occasion"
32	Ge et al.[32], 2005	BL: 0[1]<br>HA: 3685[1]	NR	10, 100%	Age, y: 34±8;<br>BMI, kg/m^2: 25±4	PaO_2, kPa: 12.8±0.93<br>$PaCO_2$, kPa: 5.6±0.4<br>SaO_2, %: NR<br>pH: NR	PaO_2, kPa: 6.8±0.8<br>$PaCO_2$, kPa: NR<br>SaO_2, %: 86±1<br>pH: NR	NR
33	Eldridge et al.[33], 2006	BL: 8[2]<br>HA: 3810[2]	ABL 520, Radiometer	8, 100%	Age, y: 30.5±5.5 range 24-40;<br>BMI, kg/m^2: NR	PaO_2, kPa: 14.13±0.38<br>$PaCO_2$, kPa: 5.28±0.65<br>SaO_2, %: NR<br>pH: NR	PaO_2, kPa: 6.27±0.75<br>$PaCO_2$, kPa: 4.61±0.23<br>SaO_2, %: NR<br>pH: NR	2
34	Ainslie et al.[34], 2007	BL: 1400[2]<br>HA: 3840[2]	i-STAT, Abbott Diagnostics	5, 60%	Age, y: 32±12;<br>BMI, kg/m^2: 23±2	PaO_2, kPa: 9.92±0.45<br>$PaCO_2$, kPa: 4.77±0.32<br>SaO_2, %: 95.4±0.9<br>pH: 7.425±0.042	PaO_2, kPa: 7.25±0.41<br>$PaCO_2$, kPa: 3.16±0.61<br>SaO_2, %: 90.2±1.8<br>pH: 7.51±0.009	0
35	Ainslie et al.[35], 2008	BL: 1400[2]<br>HA: 5400[2]	i-STAT, Abbott Diagnostics	10, 70%	Age, y: 34±11;<br>BMI, kg/m^2: 24±3	PaO_2, kPa: 9.67±0.61<br>$PaCO_2$, kPa: 4.96±0.39<br>SaO_2, %: 94.6±1.3<br>pH: 7.421±0.033	PaO_2, kPa: 9.25±0.47<br>$PaCO_2$, kPa: 3.24±0.24<br>SaO_2, %: 75±2.8<br>pH: 7.515±0.017	Yes, AMS but not how many
36	Berger et al.[36], 2009	BL: 110[2]<br>HA: 4559[2]	ABL 5, Radiometer	34, 85.3%	Age, y: 36.9±9.9;<br>BMI, kg/m^2: NR	PaO_2, kPa: 12.67±1.33<br>$PaCO_2$, kPa: NR<br>SaO_2, %: NR<br>pH: NR	PaO_2, kPa: 5.2±0.4<br>$PaCO_2$, kPa: NR<br>SaO_2, %: NR<br>pH: NR	15

© 2023 Forrer A et al. *JAMA Network Open*.

Index	Publication	Altitude [m]	Analyzer	N, % male	Demographics	Arterial blood gases at BL [kPa]	Arterial blood gases at HA [kPa]	ARAHE, overall
37	Bailey et al.[37], 2010	BL: 110[2]<br>HA: 4559[2]	ABL 5, Radiometer	36, 84.2%	Age, y: 36±10;<br>BMI, kg/m^2: NR	PaO_2, kPa: 12.67±1.47<br>$PaCO_2$, kPa: 5.33±0.4<br>SaO_2, %: 97±1<br>pH: 7.41±0.03	PaO_2, kPa: 5.07±0.4<br>$PaCO_2$, kPa: 3.87±0.4<br>SaO_2, %: 72±5<br>pH: 7.51±0.02	14
38	Fan et al.[38], 2010	BL: 0[2]<br>HA: 5050[2]	NPT 7 series, Radiometer	17, 64.7%	Age, y: 31±9;<br>BMI, kg/m^2: 23±2	PaO_2, kPa: 14.0±1.47<br>$PaCO_2$, kPa: 5.6±0.4<br>SaO_2, %: 98.4±0.05<br>pH: 7.45±0.04	PaO_2, kPa: 5.87±0.4<br>$PaCO_2$, kPa: 3.87±0.4<br>SaO_2, %: 79.9±3.4<br>pH: 7.47±0.03	4
39	Hansen et al.[39], 2012	BL: 0[1]<br>HA: 1438 / 3048 / 3658 / 4267 / 4877[1]	ABL 725, Radiometer	10, 70%	Age, y: 25 range 20-34;<br>BMI, kg/m^2: 24	PaO_2, kPa: 12.6±0.2<br>$PaCO_2$, kPa: 4.7±0.2<br>SaO_2, %: NR<br>pH: NR	PaO_2, kPa: 8.6±0.2 / 8.0±0.2 / 7.0±0.2 / 6.0±0.2 / 5.3±0.2<br>$PaCO_2$, kPa: 4.7±0.1 / 4.6±0.1 / 4.4±0.1 / 4.3±0.1 / 4.3±0.1<br>SaO_2, %: NR<br>pH: NR	1
40	Burgess et al.[40], 2013	BL: 0[2]<br>HA: 5050[2]	NPT 7 series, Radiometer	12, 66.7%	Age, y: 30±10;<br>BMI, kg/m^2: 23±3	PaO_2, kPa: 13.33±1.07<br>$PaCO_2$, kPa: 5.6±0.54<br>SaO_2, %: NR<br>pH: 7.44±0.02	PaO_2, kPa: 5.73±0.4<br>$PaCO_2$, kPa: 3.87±0.4<br>SaO_2, %: NR<br>pH: 7.47±0.03	5
41	Spliethoff et al.[41], 2013	BL: 0[2]<br>HA: 4559[2]	ABL 5, Radiometer	25, 60%	Age, y: range 20-60;<br>BMI, kg/m^2: NR	PaO_2, kPa: 12.4±1.2<br>$PaCO_2$, kPa: 5.1±0.4<br>SaO_2, %: 95.1±1.2<br>pH: 7.424±0.032	PaO_2, kPa: 5.2±0.6<br>$PaCO_2$, kPa: 3.8±0.3<br>SaO_2, %: 76.8±4.6<br>pH: 7.47	13
42	Subudhi et al.[42], 2014	BL: 130[2]<br>HA: 5050[2]	RAPIDLab 248, Siemens, OSM 3, Radiometer	21, 57.1%	Age, y: 20.8 range 19-23;<br>BMI, kg/m^2: NR	PaO_2, kPa: 13.63±0.71<br>$PaCO_2$, kPa: 5.15±0.46<br>SaO_2, %: 98.57±0.66<br>pH: 7.406±0.027	PaO_2, kPa: 4.85±0.4<br>$PaCO_2$, kPa: 3.54±0.4<br>SaO_2, %: 76.39±6.12<br>pH: 7.505±0.027	
43	Nakano et al.[43], 2015	BL: 0[1]<br>HA: 2000 / 3000 / 4000 / 4500[1]	IL-1304; Instrumentation Laboratory	7, 100%	Age, y: 23.4±9.2 range 19-46;<br>BMI, kg/m^2: 21.8±1.82	PaO_2, kPa: 12.84±0.71<br>$PaCO_2$, kPa: 5.24±0.31<br>SaO_2, %: 96±1<br>pH: 7.39±0.01	PaO_2, kPa: 9.52±1.6 / 8.27±1.37 / 6.75±0.6 / 6.24±1.07	NR

© 2023 Forrer A et al. *JAMA Network Open*.

Index	Publication	Altitude [m]	Analyzer	N, % male	Demographics	Arterial blood gases at BL [kPa]	Arterial blood gases at HA [kPa]	ARAHE, overall
							$PaCO_2$, kPa: 4.81±0.65 / 4.7±0.8 / 4.64±0.65 / 4.6±0.64<br>SaO_2, %: 93±2 / 90±3 / 86±4 / 83±5<br>pH: 7.43±0.05 / 7.43±0.06 / 7.43±0.04 / 7.44±0.04	
44	Hilty et al.[44], 2016	BL: 446[2]<br>HA: 4559[2]	ABL 800 Flex, Radiometer	17, 71%	Age, y: 44.8±9;<br>BMI, kg/m^2: 24.1±2.8	PaO_2, kPa: 11.8±1.8<br>$PaCO_2$, kPa: NR<br>SaO_2, %: 96±1<br>pH: NR	PaO_2, kPa: 5.3±0.3<br>$PaCO_2$, kPa: NR<br>SaO_2, %: 76±4<br>pH: NR	9
45	Leacy et al.[45], 2018	BL: 1045[2]<br>HA: 3440 / 3820 / 4240[2]	i-Stat, Abbott Diagnostics	10	Age, y: 21.7±1.3;<br>BMI, kg/m^2: 23.57±2	PaO_2, kPa: 11.31±0.71<br>$PaCO_2$, kPa: 4.64±0.61<br>SaO_2, %: 96.8±0.79<br>pH: 7.43±0.02	PaO_2, kPa: 6.47±1.08 / 7.27±0.83 / 6.45±0.66<br>$PaCO_2$, kPa: 4.03±0.66 / 3.94±0.5 / 3.87±0.5<br>SaO_2, %: 84.89±7.87 / 88.78±3.73 / 85.5±3.66<br>pH: 7.45±0.02 / 7.43±0.02 / 7.44±0.02	1
46	Wolff et al.[46], 2018	BL: 550[2]<br>HA: 4844 / 6022 / 7050[2]	Epoc System, Alere	40, 52.5%	Age, y: 45.56±12.06;<br>BMI, kg/m^2: NR	PaO_2, kPa: 12.71±1.05<br>$PaCO_2$, kPa: NR<br>SaO_2, %: 97.57±0.79<br>pH: NR	PaO_2, kPa: 5.89±0.6 / 4.38±0.67 / 4.05±0.59<br>$PaCO_2$, kPa: NR<br>SaO_2, %: 83.78±4.59 / 72.71±8.83 / 68.8±9.65<br>pH: NR	NR
47	Zouboules et al.[47], 2018	BL: 1045[2]<br>HA: 3440 / 3820 / 4240 / 5160[2]	i-STAT, Abbott Diagnostics	20, 50%	Age, y: 27.5 range 19-49;<br>BMI, kg/m^2: 26.1±4.4	PaO_2, kPa: 10.69±1.11<br>$PaCO_2$, kPa: 4.76±0.51<br>SaO_2, %: 96±1.5<br>pH: 7.43±0.02	PaO_2, kPa: 6.52±0.95 / 6.99±0.76 / 6.39±0.67 / 4.79±0.64<br>$PaCO_2$, kPa: 4.09±0.51 / 4.04±0.44 / 3.87±0.43 / 3.44±0.32	NR

© 2023 Forrer A et al. *JAMA Network Open*.

Index	Publication	Altitude [m]	Analyzer	N, % male	Demographics	Arterial blood gases at BL [kPa]	Arterial blood gases at HA [kPa]	ARAHE, overall
							SaO_2, %: 85.4±6.4 / 87.6±3.4 / 85.2±3.9 / 72.9±7.2<br>pH: 7.451±0.025 / 7.432±0.022 / 7.441±0.022 / 7.459±0.018	
48	Hilty et al.[48], 2019	BL: 553[2]<br>HA: 6022 / 7042[2]	Epoc System; Alere	36, 54%	Age, y: 45.8±11.9;<br>BMI, kg/m^2: NR	PaO_2, kPa: 12.7±1.1<br>$PaCO_2$, kPa: 5.1±0.4<br>SaO_2, %: 97.6±0.8<br>pH: 7.44±0.02	PaO_2, kPa: 4.6±0.7 / 4.1±0.6<br>$PaCO_2$, kPa: 2.8±0.3 / 1.8±0.3<br>SaO_2, %: 72.7±8.8 / 65.8±14.2<br>pH: 7.5±0.02 / 7.53±0.06	9
49	Hoiland et al.[49], 2019	BL: 1400[2]<br>HA: 3400 / 4371 / 5050[2]	i-STAT 1, Abbott Diagnostics	21, 95.24%	Age, y: 28±6;<br>BMI, kg/m^2: 23±2	PaO_2, kPa: 10.27±0.87<br>$PaCO_2$, kPa: 5.37±0.35<br>SaO_2, %: 95.4±1.2<br>pH: 7.42±0.02	PaO_2, kPa: 6.91±0.55 / 6.35±0.49 / 5.49±0.59<br>$PaCO_2$, kPa: 4.6±0.19 / 4.29±0.21 / 3.99±0.21<br>SaO_2, %: 87.4±2.7 / 84.5±3.2 / 78.95±4.94<br>pH: 7.44±0.02 / 7.43±0.02 / 7.46±0.02	NR
50	Xie et al.[50], 2019	BL: 0[1]<br>HA: 4000[1]	IL-1304; Instrumentation Laboratory	25, 56%	Age, y: 26.6±2.3 range 21-33;<br>BMI, kg/m^2: NR	PaO_2, kPa: 14.92±1.87<br>$PaCO_2$, kPa: 4.68±0.38<br>SaO_2, %: 97.61±0.48<br>pH: 7.4±0.02	PaO_2, kPa: 5.87±0.99<br>$PaCO_2$, kPa: 4.23±0.51<br>SaO_2, %: 80.95±6.68<br>pH: 7.42±0.03	NR
51	Steele et al.[51], 2020	BL: 344[2]<br>HA: 4330[2]	i-STAT, Abbott Diagnostics	24, 83.3%	Age, y: 28±6.4;<br>BMI, kg/m^2: 24.3±2.4	PaO_2, kPa: 13.41±2.45<br>$PaCO_2$, kPa: 5.12±0.43<br>SaO_2, %: 97.61±1.2<br>pH: 7.43±0.033	PaO_2, kPa: 5.53±0.97<br>$PaCO_2$, kPa: 4.41±0.44<br>SaO_2, %: 78.9±8.4<br>pH: 7.48±0.034	13
52	Bird et al.[52], 2021	BL: 1045[2]<br>HA: 3800[2]	i-STAT, Abbott Diagnostics	16, 68.75%	Age, y : 31.3±11.7<br>BMI, kg/m^2: 26.0±3.8	PaO_2, kPa: 10.78±1.17<br>$PaCO_2$, kPa: 5.12±0.4<br>SaO_2, %: 95.8±1.2	PaO_2, kPa: 6.73±0.69<br>$PaCO_2$, kPa: 4.57±0.37<br>SaO_2, %: 85.8±3.8	NR

© 2023 Forrer A et al. *JAMA Network Open*.

Index	Publication	Altitude [m]	Analyzer	N, % male	Demographics	Arterial blood gases at BL [kPa]	Arterial blood gases at HA [kPa]	ARAHE, overall
						pH: 7.412±0.014	pH: 7.424±0.017	
53	Furian et al.[53], 2022	BL: 760[2]<br>HA: 3100[2]	Rapidpoint 500, Siemens	109, 30%	Age, y: 54±7;<br>BMI, kg/m^2: 27.8±4	PaO_2, kPa: 10.13±0.13<br>$PaCO_2$, kPa: 5.2±0<br>SaO_2, %: 94.8±0.1<br>pH: 7.4±0	PaO_2, kPa: 7.73±0.13<br>$PaCO_2$, kPa: 4.67±0<br>SaO_2, %: 88.4±0.2<br>pH: 7.43±0	54 (of 161 at HA)
	Values presented in numbers or mean ±SD or median (quartiles). Standard error of the mean (SE) can be calculated as SE = SD / $\sqrt{\text{sample size}}$. [1]hypobaric chamber study. [2]high-altitude study. BL = baseline; HA = high altitude; NR = not reported; ARAHE = altitude-related adverse health effects; AMS = acute mountain sickness; $PaCO_2$ / O_2 = arterial partial pressure of oxygen and carbon dioxide; SaO_2 = arterial oxygen saturation.							

© 2023 Forrer A et al. *JAMA Network Open*.

eTable 2. Baseline Factors Associated With PaO_2 at Altitude Based on Individual Participant Data

Predictor	*Coefficient*	*Standard Error*	*P-Value*	*95% Confidence Interval*
Intercept	13.185	0.596	<0.001	12.016 to 14.354
Target altitude [km]	-1.525	0.053	<0.001	-1.629 to -1.421
Age [years]	-0.013	0.005	0.010	-0.023 to -0.003
Female vs Male	0.122	1.000	0.223	-0.074 to 0.318
PaO_2 at low altitude [kPa]	-0.035	0.034	0.309	-0.101 to 0.032
Time above 1500m [day]	0.163	0.025	<0.001	0.114 to 0.213

The equation based on the regression model is the following: $PaO_{2_{altitude}}[kPa] = 13.185 - 1.525 \times target\ altitude\ [km] - 0.013 \times age\ [yrs] + 0.122 \times gender\ [0 = male;\ 1 = female] - 0.035 \times PaO_{2_{low\ altitude}}[kPa] + 0.163 \times time\ above\ 1500m\ [days]$. PaO_2: arterial partial pressure of oxygen. This model was determined with the Akaike and Bayesian Information Criterion. The model did not further improve when removing PaO_2 at low altitude or any other variable.

© 2023 Forrer A et al. *JAMA Network Open*.

eTable 3. Exploratory Regression Analysis of Individual Participant Data Using Log PaO_2 as Dependent Variable

Predictor	***Coefficient***	***Standard Error***	***P-Value***	***95% Confidence Interval***
Intercept	2.796	0.092	<0.001	2.616 to 2.977
Target altitude [km]	-0.214	0.000	<0.001	-0.230 to -0.199
Age [years]	-0.001	0.001	0.084	-0.003 to 0.000
Female vs Male	0.023	0.015	0.131	-0.007 to 0.054
PaO_2 at low altitude [kPa]	-0.007	0.005	0.167	-0.018 to 0.003
Time above 1500m [day]	0.022	0.004	<0.001	0.015 to 0.030

PaO_2: arterial partial pressure of oxygen

© 2023 Forrer A et al. *JAMA Network Open*.

eTable 4. Risk of Bias Assessment

Index	Title	1. Was the research question or objective in this paper clearly stated?	2. Was the study population clearly specified and defined?	3. Was the participation rate of eligible persons at least 50%?	4. Were all the subjects selected or recruited from the same or similar populations (including the same time period)? Were inclusion and exclusion criteria for being in the study prespecified and applied uniformly to all participants?	5. Was a sample size justification, power description, or variance and effect estimates provided?	6. For the analyses in this paper, were the exposure(s) of interest measured prior to the outcome(s) being measured?	7. Was the timeframe sufficient so that one could reasonably expect to see an association between exposure and outcome if it existed?	8. For exposures that can vary in amount or level, did the study examine different levels of the exposure as related to the outcome?	9. Were the exposure measures (independent variables) clearly defined, valid, reliable, and implemented consistently across all study participants?	10. Was the exposure(s) assessed more than once over time?	11. Were the outcome measures (dependent variables) clearly defined, valid, reliable, and implemented consistently across all study participants?	12. Were the outcome assessors blinded to the exposure status of participants?	13. Was loss to follow-up after baseline 20% or less?	14. Were key potential confounding variables measured and adjusted statistically for their impact on the relationship between exposure(s) and outcome(s)?	Quality rating
1	Kann et al.[1], 1967	1	1	NR	1	0	1	1	1	0	1	0	0	0/1/1/0	0	poor
2	Reeves et al.[2], 1967	1	1	NR	1	0	1	1	0	1	1	1	0	1	0	fair
3	Vogel et al.[3], 1967	1	1	NR	1	0	1	1	1	1	1	1	0	1	0	fair
4	Reeves et al.[4], 1969	1	1	NR	1	0	1	1	0	1	1	1	0	1	0	fair
5	Moncloa et al.[5], 1970	1	0	NR	1	0	1	1	0	1	1	1	0	1	0	fair
6	Dempsey et al.[6], 1974	1	1	NR	1	0	1	1	1	1	1	1	0	1	0	fair
7	Hartmann et al.[7], 1974	1	0	0	1	0	1	1	1	1	1	1	0	1	0	fair
8	Vogel et al.[8], 1974	1	1	NR	1	0	1	1	0	1	1	1	0	1	0	fair

© 2023 Forrer A et al. *JAMA Network Open*.

9	Cruz et al.[9], 1975	1	1	NR	1	0	1	1	1	1	1	1	0	1	0	fair
10	Frayser et al.[10], 1975	1	0	NR	NR	0	1	1	1	1	1	1	0	1	0	fair
11	Evans et al.[11], 1976	1	1	NR	1	0	1	1	0	1	1	1	0	1	0	fair
12	Hannon et al.[12], 1977	1	1	NR	1	0	1	1	0	1	1	1	0	1	0	fair
13	Torre-Bueno et al.[13], 1985	1	0	NR	NR	0	NR	NR	1	0	NR	1	0	1	0	poor
14	Wagner et al.[14], 1986	1	0	NR	1	0	1	1	1	1	1	1	0	1	0	fair
15	Bender et al.[15], 1988	1	0	NR	1	0	1	1	1	1	1	1	0	1	0	fair
16	Sutton et al.[16], 1988	1	1	0	1	0	1	1	1	1	1	1	0	1/0/0/0	0	fair
17	Kawashima et al.[17], 1989	1	1	NR	1	0	1	1	0	1	1	1	0	1	0	fair
18	Bärtsch et al.[18], 1991	1	0	NR	1	0	1	1	1	1	1	1	0	1	0	fair
19	Wolfel et al.[19], 1991	1	1	NR	1	0	1	1	0	1	1	1	0	1	0	fair
20	Levine et al.[20], 1992	1	1	NR	1	0	1	1	1	1	1	1	0	1	0	fair
21	Dillard et al.[21], 1995	1	1	NR	1	0	1	1	0	1	1	1	0	1	0	fair
22	Goerre et al.[22], 1995	1	1	NR	1	0	1	1	1	1	1	1	0	1/1/1/0	0	fair
23	Podolsky et al.[23], 1996	1	1	NR	1	0	1	1	0	0	1	1	0	1	0	poor
24	Young et al.[24], 1996	1	1	NR	1	0	1	1	1	1	1	1	0	1	0	fair
25	Dillard et al.[25], 1998	1	1	NR	1	0	1	1	0	1	1	1	0	1	0	fair
26	Wolfel et al.[26], 1998	1	1	NR	1	0	1	1	0	1	1	1	0	1	0	fair
27	Imoberdorf et al.[27], 2001	1	1	NR	1	0	1	1	1	1	1	0	0	1	0	poor
28	Maggiorini et al.[28], 2001	1	0	NR	1	0	1	1	NR	1	1	1	0	1	0	fair
29	Imray et al.[29], 2003	1	1	NR	1	0	1	1	0	1	1	1	0	1	0	fair
30	Mairbäurl et al.[30], 2003	1	0	NR	1	0	1	1	1	1	1	1	0	1	0	fair
31	Burgess et al.[31], 2004	1	0	NR	1	0	1	1	1	1	1	1	0	1	0	fair
32	Ge et al.[32], 2005	1	1	NR	1	0	1	1	0	0	1	0	0	1	0	poor
33	Eldridge et al.[33], 2006	1	1	NR	1	0	1	1	0	1	1	1	0	1	0	fair
34	Ainslie et al.[34], 2007	1	1	NR	1	0	1	1	0	0	1	1	0	1	0	poor
35	Ainslie et al.[35], 2008	1	1	NR	1	0	1	1	0	0	1	1	0	1	0	poor
36	Berger et al.[36], 2009	1	1	NR	1	0	1	1	1	1	1	1	0	1	0	fair
37	Bailey et al.[37], 2010	1	1	NR	1	0	1	1	1	1	1	1	0	0	0	fair

© 2023 Forrer A et al. *JAMA Network Open*.

38	Fan et al.[38], 2010	1	1	NR	1	0	1	1	0	1	1	1	0	1	0	fair
39	Hansen et al.[39], 2012	1	1	NR	1	0	1	1	1	1	1	1	0	1	0	fair
40	Burgess et al.[40], 2013	1	1	NR	1	0	1	1	1	1	1	1	0	1	0	fair
41	Spliethoff et al.[41], 2013	1	1	NR	1	1	1	1	0	1	1	1	0	1	0	fair
42	Subudhi et al.[42], 2014	1	1	0	1	0	1	1	0	1	1	1	0	1	0	fair
43	Nakano et al.[43], 2015	1	1	NR	1	0	1	1	1	1	1	1	0	1	0	fair
44	Hilty et al.[44], 2016	1	1	NR	1	0	1	1	0	1	1	1	0	1	0	fair
45	Leacy et al.,[45] 2018	1	1	NR	1	0	1	1	1	1	1	1	0	1	0	fair
46	Wolff et al.[46], 2018	1	1	NR	1	0	1	1	1	1	1	1	0	1/1/0/0	0	fair
47	Zouboules et al.[47], 2018	1	1	NR	1	0	1	1	1	1	1	1	0	1	0	fair
48	Hilty et al.[48], 2019	1	1	NR	1	0	1	1	1	1	1	1	0	1/0	0	fair
49	Hoiland et al.[49], 2019	1	1	NR	1	0	1	1	1	1	1	1	0	1	0	fair
50	Xie et al.[50], 2019	1	1	NR	1	0	1	1	0	1	1	1	0	1	0	fair
51	Steele et al.[51], 2020	1	1	NR	1	0	1	1	0	1	1	1	0	1	0	fair
52	Bird et al.[52], 2021	1	1	NR	1	0	1	1	1	1	1	1	0	1	0	fair
53	Furian et al.[53], 2022	1	1	0	1	0	1	1	0	1	1	1	0	1	0	fair

© 2023 Forrer A et al. *JAMA Network Open*.

eFigure 1. Galbraith Plot

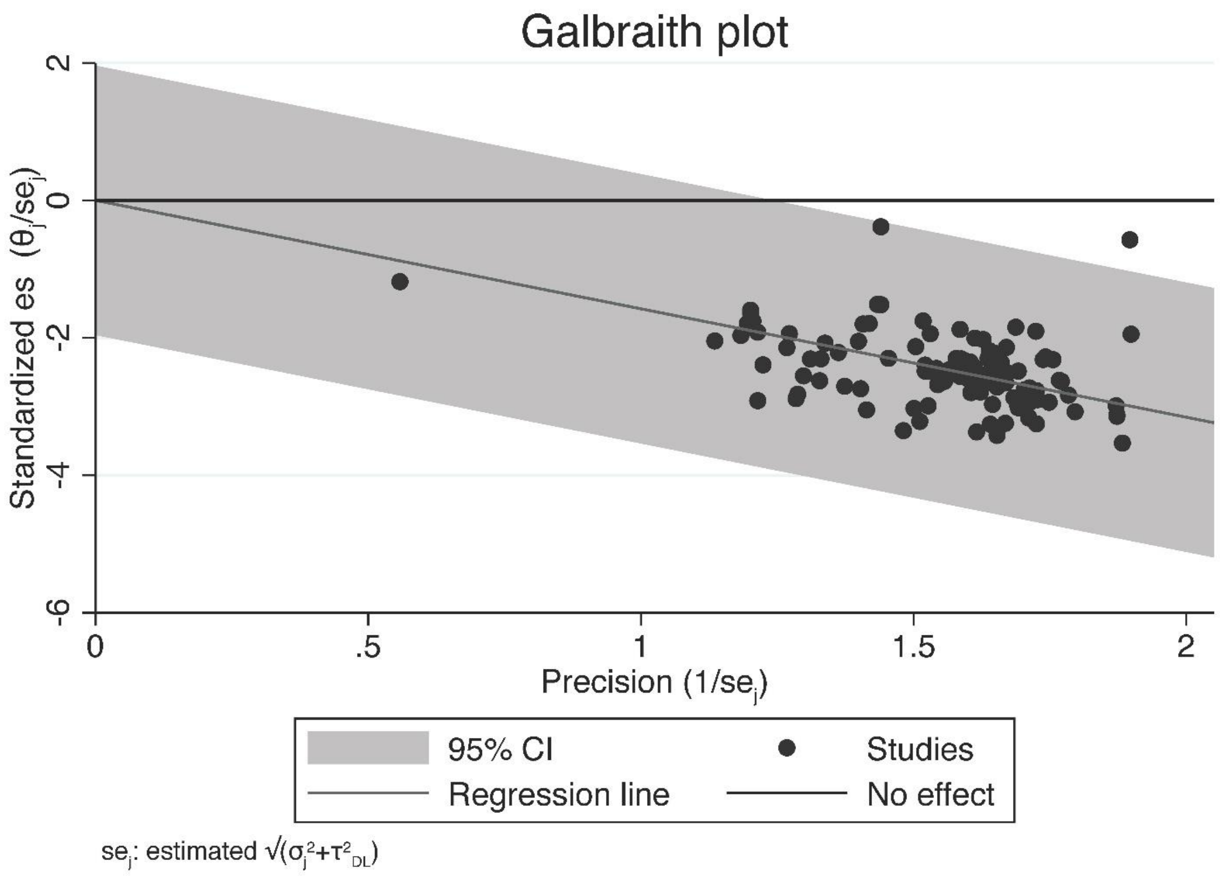

Galbraith plot. With this plot, we analyzed heterogeneity across the studies. Three studies were far out from the others and based on this visual interpretation as well as medical plausibility they were excluded.

© 2023 Forrer A et al. *JAMA Network Open*.

eFigure 2. Funnel Plot

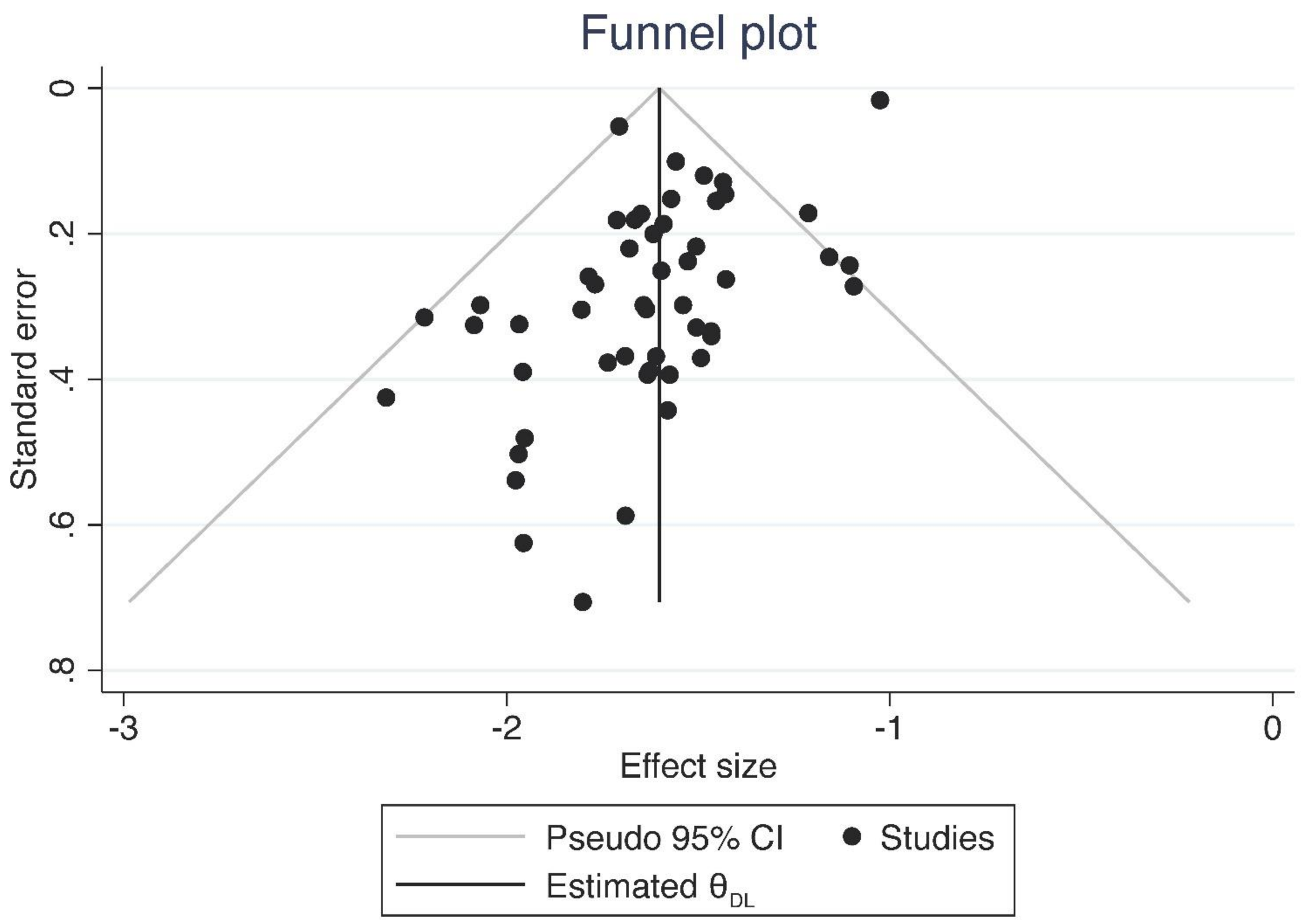

Funnel plot. Funnel plot for checking the publication bias.

© 2023 Forrer A et al. *JAMA Network Open*.

eFigure 3. Arterial Blood Gas Values Expressed by Percentage From Low Altitude

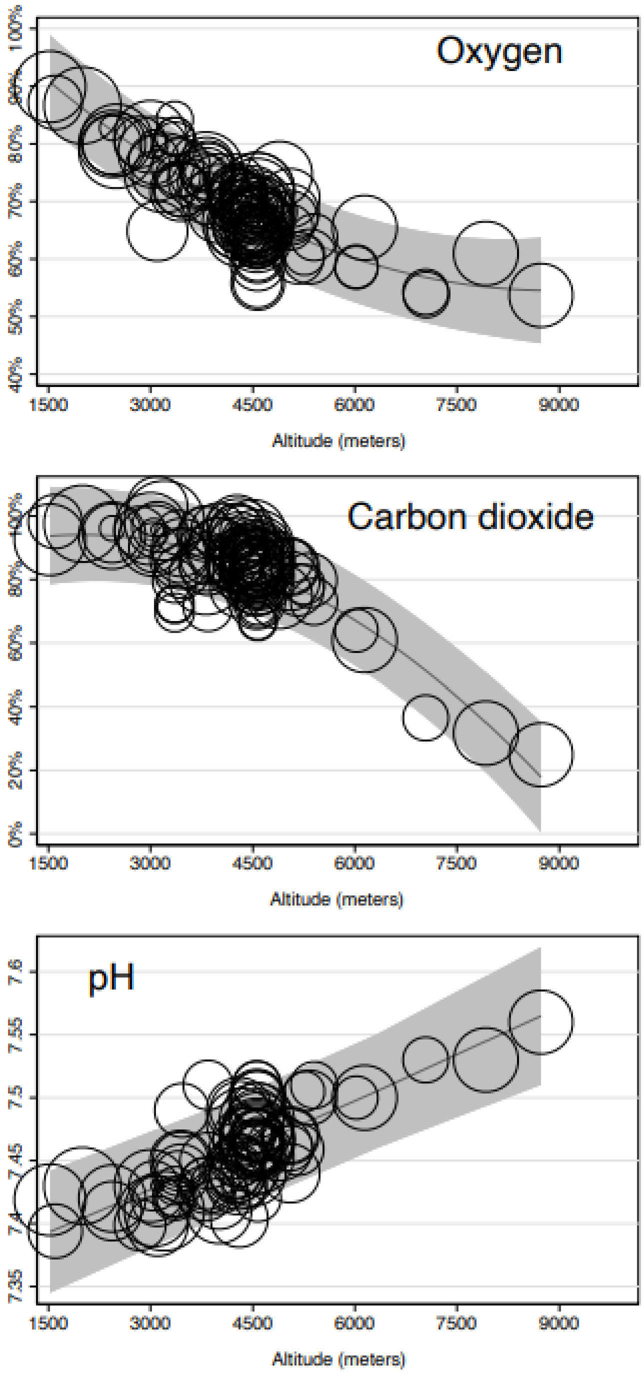

For this analysis the log-value of arterial blood gas results are expressed as a function from baseline. The baseline value (100%) is 13.3 kPa for PaO_2 and 5.0 for $PaCO_2$, which is the average at 0 meters based on 16 studies.[5,8,13-16,19-21,23-26,31,32,38,43,50]

© 2023 Forrer A et al. *JAMA Network Open*.

eReferences

1. Kann J, Szadkowski D, Obermayer-Beckmann H. The behavior of blood gases in various altitudes above sea level and its significance for investigations of occupational medicine. Article. *Internationales Archiv für Arbeitsmedizin*. 1967;23(3):270-280.
2. Reeves JT, Grover RF, Cohn JE. Regulation of ventilation during exercise at 10,200 ft in athletes born at low altitude. *J Appl Physiol*. Mar 1967;22(3):546-54. doi:10.1152/jappl.1967.22.3.546
3. Vogel JA, Harris CW. Cardiopulmonary responses of resting man during early exposure to high altitude. *J Appl Physiol*. Jun 1967;22(6):1124-8. doi:10.1152/jappl.1967.22.6.1124
4. Reeves JT, Halpin J, Cohn JE, Daoud F. Increased alveolar-arterial oxygen difference during simulated high-altitude exposure. *J Appl Physiol*. Nov 1969;27(5):658-61. doi:10.1152/jappl.1969.27.5.658
5. Moncloa F, Carcelen A, Beteta L. Physical exercise, acid-base balance, and adrenal function in newcomers to high altitude. *J Appl Physiol*. Feb 1970;28(2):151-5. doi:10.1152/jappl.1970.28.2.151
6. Dempsey JA, Forster HV, DoPico GA. Ventilatory acclimatization to moderate hypoxemia in man. The role of spinal fluid (H+). *J Clin Invest*. Apr 1974;53(4):1091-100. doi:10.1172/jci107646
7. Hartmann B, Unger M, Debelić M, Hilpert P. [Arterial blood gas tensions and airways obstruction in normal subjects and patients with obstructive lung disease before and during adaptation to altitude (author's transl)]. *Respiration*. Jan 1974;31(1):7-20. Blutgaswerte und Atemwegswiderstand bei Gesunden und Patienten mit obstruktiven Atemwegserkrankungen nor und nach Höhenadaptation. doi:10.1159/000193095
8. Vogel JA, Hartley LH, Cruz JC, Hogan RP. Cardiac output during exercise in sea-level residents at sea level and high altitude. *J Appl Physiol*. Feb 1974;36(2):169-72. doi:10.1152/jappl.1974.36.2.169
9. Cruz JC, Hartley LH, Vogel JA. Effect of altitude relocations upon AaDo2 at rest and during exercise. *J Appl Physiol*. Sep 1975;39(3):469-74. doi:10.1152/jappl.1975.39.3.469
10. Frayser R, Rennie ID, Gray GW, Houston CS. Hormonal and electrolyte response to exposure to 17,500 ft. *J Appl Physiol*. Apr 1975;38(4):636-42. doi:10.1152/jappl.1975.38.4.636
11. Evans WO, Robinson SM, Horstman DH, Jackson RE, Weiskopf RB. Amelioration of the symptoms of acute mountain sickness by staging and acetazolamide. *Aviat Space Environ Med*. May 1976;47(5):512-6.
12. Hannon JP, Vogel JA. Oxygen transport during early altitude acclimatization: a perspective study. *Eur J Appl Physiol Occup Physiol*. May 10 1977;36(4):285-97. doi:10.1007/bf00423054
13. Torre-Bueno JR, Wagner PD, Saltzman HA, Gale GE, Moon RE. Diffusion limitation in normal humans during exercise at sea level and simulated altitude. *J Appl Physiol (1985)*. Mar 1985;58(3):989-95. doi:10.1152/jappl.1985.58.3.989
14. Wagner PD, Gale GE, Moon RE, Torre-Bueno JR, Stolp BW, Saltzman HA. Pulmonary gas exchange in humans exercising at sea level and simulated altitude. *J Appl Physiol (1985)*. Jul 1986;61(1):260-70. doi:10.1152/jappl.1986.61.1.260
15. Bender PR, Groves BM, McCullough RE, et al. Oxygen transport to exercising leg in chronic hypoxia. *J Appl Physiol (1985)*. Dec 1988;65(6):2592-7. doi:10.1152/jappl.1988.65.6.2592
16. Sutton JR, Reeves JT, Wagner PD, et al. Operation Everest II: oxygen transport during exercise at extreme simulated altitude. *J Appl Physiol (1985)*. Apr 1988;64(4):1309-21. doi:10.1152/jappl.1988.64.4.1309
17. Kawashima A, Kubo K, Kobayashi T, Sekiguchi M. Hemodynamic responses to acute hypoxia, hypobaria, and exercise in subjects susceptible to high-altitude pulmonary edema. *J Appl Physiol (1985)*. Nov 1989;67(5):1982-9. doi:10.1152/jappl.1989.67.5.1982
18. Bärtsch P, Maggiorini M, Ritter M, Noti C, Vock P, Oelz O. Prevention of high-altitude pulmonary edema by nifedipine. *N Engl J Med*. Oct 31 1991;325(18):1284-9. doi:10.1056/nejm199110313251805
19. Wolfel EE, Groves BM, Brooks GA, et al. Oxygen transport during steady-state submaximal exercise in chronic hypoxia. *J Appl Physiol (1985)*. Mar 1991;70(3):1129-36. doi:10.1152/jappl.1991.70.3.1129
20. Levine BD, Friedman DB, Engfred K, et al. The effect of normoxic or hypobaric hypoxic endurance training on the hypoxic ventilatory response. *Med Sci Sports Exerc*. Jul 1992;24(7):769-75.

© 2023 Forrer A et al. *JAMA Network Open*.

21. Dillard TA, Moores LK, Bilello KL, Phillips YY. The preflight evaluation. A comparison of the hypoxia inhalation test with hypobaric exposure. *Chest*. Feb 1995;107(2):352-7. doi:10.1378/chest.107.2.352
22. Goerre S, Wenk M, Bärtsch P, et al. Endothelin-1 in pulmonary hypertension associated with high-altitude exposure. *Circulation*. Jan 15 1995;91(2):359-64. doi:10.1161/01.cir.91.2.359
23. Podolsky A, Eldridge MW, Richardson RS, et al. Exercise-induced VA/Q inequality in subjects with prior high-altitude pulmonary edema. *J Appl Physiol (1985)*. Aug 1996;81(2):922-32. doi:10.1152/jappl.1996.81.2.922
24. Young AJ, Sawka MN, Muza SR, et al. Effects of erythrocyte infusion on VO2max at high altitude. *J Appl Physiol (1985)*. Jul 1996;81(1):252-9. doi:10.1152/jappl.1996.81.1.252
25. Dillard TA, Rajagopal KR, Slivka WA, Berg BW, Mehm WJ, Lawless NP. Lung function during moderate hypobaric hypoxia in normal subjects and patients with chronic obstructive pulmonary disease. *Aviat Space Environ Med*. Oct 1998;69(10):979-85.
26. Wolfel EE, Selland MA, Cymerman A, et al. O2 extraction maintains O2 uptake during submaximal exercise with beta-adrenergic blockade at 4,300 m. *J Appl Physiol (1985)*. Sep 1998;85(3):1092-102. doi:10.1152/jappl.1998.85.3.1092
27. Imoberdorf R, Garlick PJ, McNurlan MA, et al. Enhanced synthesis of albumin and fibrinogen at high altitude. *J Appl Physiol (1985)*. Feb 2001;90(2):528-37. doi:10.1152/jappl.2001.90.2.528
28. Maggiorini M, Mélot C, Pierre S, et al. High-altitude pulmonary edema is initially caused by an increase in capillary pressure. *Circulation*. Apr 24 2001;103(16):2078-83. doi:10.1161/01.cir.103.16.2078
29. Imray CH, Walsh S, Clarke T, et al. Effects of breathing air containing 3% carbon dioxide, 35% oxygen or a mixture of 3% carbon dioxide/35% oxygen on cerebral and peripheral oxygenation at 150 m and 3459 m. *Clin Sci (Lond)*. Mar 2003;104(3):203-10. doi:10.1042/cs20020102
30. Mairbäurl H, Weymann J, Möhrlein A, et al. Nasal epithelium potential difference at high altitude (4,559 m): evidence for secretion. *Am J Respir Crit Care Med*. Mar 15 2003;167(6):862-7. doi:10.1164/rccm.200208-864OC
31. Burgess KR, Johnson P, Edwards N, Cooper J. Acute mountain sickness is associated with sleep desaturation at high altitude. *Respirology*. Nov 2004;9(4):485-92. doi:10.1111/j.1440-1843.2004.00625.x
32. Ge RL, Stone JA, Levine BD, Babb TG. Exaggerated respiratory chemosensitivity and association with SaO2 level at 3568 m in obesity. *Respir Physiol Neurobiol*. Mar 2005;146(1):47-54. doi:10.1016/j.resp.2004.11.009
33. Eldridge MW, Braun RK, Yoneda KY, Walby WF. Effects of altitude and exercise on pulmonary capillary integrity: Evidence for subclinical high-altitude pulmonary edema. Article. *Journal of Applied Physiology*. 2006;100(3):972-980. doi:10.1152/japplphysiol.01048.2005
34. Ainslie PN, Burgess K, Subedi P, Burgess KR. Alterations in cerebral dynamics at high altitude following partial acclimatization in humans: wakefulness and sleep. *J Appl Physiol (1985)*. Feb 2007;102(2):658-64. doi:10.1152/japplphysiol.00911.2006
35. Ainslie PN, Ogoh S, Burgess K, et al. Differential effects of acute hypoxia and high altitude on cerebral blood flow velocity and dynamic cerebral autoregulation: alterations with hyperoxia. *J Appl Physiol (1985)*. Feb 2008;104(2):490-8. doi:10.1152/japplphysiol.00778.2007
36. Berger MM, Dehnert C, Bailey DM, et al. Transpulmonary plasma ET-1 and nitrite differences in high altitude pulmonary hypertension. *High Alt Med Biol*. Spring 2009;10(1):17-24. doi:10.1089/ham.2008.1053
37. Bailey DM, Dehnert C, Luks AM, et al. High-altitude pulmonary hypertension is associated with a free radical-mediated reduction in pulmonary nitric oxide bioavailability. *J Physiol*. Dec 1 2010;588(Pt 23):4837-47. doi:10.1113/jphysiol.2010.194704
38. Fan JL, Burgess KR, Basnyat R, et al. Influence of high altitude on cerebrovascular and ventilatory responsiveness to CO2. *J Physiol*. Feb 1 2010;588(Pt 3):539-49. doi:10.1113/jphysiol.2009.184051
39. Hansen TA, Kåsin JI, Edvardsen A, Christensen CC, Wagstaff AS. Arterial oxygen pressure following whole-body vibration at altitude. *Aviat Space Environ Med*. Apr 2012;83(4):431-5. doi:10.3357/asem.3195.2012
40. Burgess KR, Lucas SJE, Shepherd K, et al. Worsening of central sleep apnea at high altitude - A role for cerebrovascular function. Article. *Journal of Applied Physiology*. 2013;114(8):1021-1028. doi:10.1152/japplphysiol.01462.2012
41. Spliethoff K, Meier D, Aeberli I, et al. Reduced insulin sensitivity as a marker for acute mountain sickness? *High Alt Med Biol*. Sep 2013;14(3):240-50. doi:10.1089/ham.2012.1128

© 2023 Forrer A et al. *JAMA Network Open*.

42. Subudhi AW, Bourdillon N, Bucher J, et al. AltitudeOmics: the integrative physiology of human acclimatization to hypobaric hypoxia and its retention upon reascent. *PLoS One*. 2014;9(3):e92191. doi:10.1371/journal.pone.0092191
43. Nakano T, Iwazaki M, Sasao G, et al. Hypobaric hypoxia is not a direct dyspnogenic factor in healthy individuals at rest. *Respir Physiol Neurobiol*. Nov 2015;218:28-31. doi:10.1016/j.resp.2015.07.009
44. Hilty MP, Zügel S, Schoeb M, Auinger K, Dehnert C, Maggiorini M. Soluble Urokinase-Type Plasminogen Activator Receptor Plasma Concentration May Predict Susceptibility to High Altitude Pulmonary Edema. *Mediators Inflamm*. 2016;2016:1942460. doi:10.1155/2016/1942460
45. Leacy JK, Zouboules SM, Nysten H, et al. The neurovascular coupling response remains intact during incremental ascent to high altitude (4370m) in acclimatized healthy volunteers. Conference Abstract. *FASEB Journal*. 2018;32(1)
46. Wolff MV, Nakas CT, Tobler M, et al. Adrenal, thyroid and gonadal axes are affected at high altitude. Article. *Endocrine Connections*. 2018;7(10):1081-1089. doi:10.1530/EC-18-0242
47. Zouboules SM, Lafave HC, O'Halloran KD, et al. Renal reactivity: acid-base compensation during incremental ascent to high altitude. *J Physiol*. Dec 2018;596(24):6191-6203. doi:10.1113/jp276973
48. Hilty MP, Merz TM, Hefti U, Ince C, Maggiorini M, Pichler Hefti J. Recruitment of non-perfused sublingual capillaries increases microcirculatory oxygen extraction capacity throughout ascent to 7126 m. *J Physiol*. May 2019;597(10):2623-2638. doi:10.1113/jp277590
49. Hoiland RL, Howe CA, Carter HH, et al. UBC-Nepal expedition: phenotypical evidence for evolutionary adaptation in the control of cerebral blood flow and oxygen delivery at high altitude. *J Physiol*. Jun 2019;597(12):2993-3008. doi:10.1113/jp277596
50. Xie Y, Yang Y, Han Y, et al. Association between arterial blood gas variation and intraocular pressure in healthy subjects exposed to acute short-term hypobaric hypoxia. Article. *Translational Vision Science and Technology*. 2019;8(6)doi:10.1167/tvst.8.6.22
51. Steele AR, Tymko MM, Meah VL, et al. Global REACH 2018: renal oxygen delivery is maintained during early acclimatization to 4,330 m. *Am J Physiol Renal Physiol*. Dec 1 2020;319(6):F1081-f1089. doi:10.1152/ajprenal.00372.2020
52. Bird JD, Leacy JK, Foster GE, et al. Time course and magnitude of ventilatory and renal acid-base acclimatization following rapid ascent to and residence at 3,800 m over nine days. *J Appl Physiol (1985)*. Jun 1 2021;130(6):1705-1715. doi:10.1152/japplphysiol.00973.2020
53. Furian M, Mademilov M, Buergin A, et al. Acetazolamide to Prevent Adverse Altitude Effects in COPD and Healthy Adults. *NEJM Evidence*. 2022;1(1):EVIDoa2100006. doi:doi:10.1056/EVIDoa2100006

© 2023 Forrer A et al. *JAMA Network Open*.
