# Supplementary material for: Partial Pressure of Arterial Oxygen in Healthy Adults at High Altitudes: A Systematic Review and Meta-Analysis
Source: JAMA Netw Open. 2023 Jun 16;6(6):e2318036. doi: 10.1001/jamanetworkopen.2023.18036 (PMC10276310; doi:10.1001/jamanetworkopen.2023.18036)
Supplement: Supplement 2. — Data Sharing Statement [file jamanetwopen-e2318036-s002.pdf]

Data Sharing Statement

Forrer. Partial Pressure of Arterial Oxygen in Healthy Adults at High Altitudes. *JAMA Netw Open*. Published June 16, 2023. doi:10.1001/jamanetworkopen.2023.18036

Data
Data available: No

Additional Information
Explanation for why data not available: This meta-analysis is not an original article.
